# The RNA binding protein IGF2BP2/IMP2 alters the cargo of cancer cell-derived extracellular vesicles supporting tumor-associated macrophages

**DOI:** 10.1186/s12964-024-01701-y

**Published:** 2024-06-27

**Authors:** Vida Mashayekhi, Annika Schomisch, Sari Rasheed, Ernesto Aparicio-Puerta, Timo Risch, Daniela Yildiz, Marcus Koch, Simon Both, Nicole Ludwig, Thierry M. Legroux, Andreas Keller, Rolf Müller, Gregor Fuhrmann, Jessica Hoppstädter, Alexandra K. Kiemer

**Affiliations:** 1 Department of Pharmacy, Pharmaceutical Biology, Saarland University, 66123 Saarbrücken, Germany; 2 Helmholtz Institute for Pharmaceutical Research Saarland (HIPS) - Helmholtz Centre for Infection Research (HZI), and Department of Pharmacy, Saarland University, Saarbrücken, Germany; 3 German Centre for Infection Research (DZIF), Brunswick, Germany; 4 Chair for Clinical Bioinformatics, Saarland University, University Hospital, Saarbrücken, Germany; 5 Institute of Experimental and Clinical Pharmacology and Toxicology, PZMS, ZHMB, Saarland University, Homburg, Germany; 6 INM - Leibniz Institute for New Materials, Saarbrücken, Germany; 7 Department of Human Genetics, Saarland University, Homburg, Germany; 8 Department of Pharmacy, Saarland University, Saarbrücken, Germany; 9 Department of Pharmaceutical Biology, Friedrich-Alexander-Universität Erlangen-Nürnberg, Erlangen, Germany

**Keywords:** Flow cytometry, Tangential flow filtration, Ultracentrifugation, MMP9, Colorectal cancer, microRNAs, EV uptake inhibitors, Phagocytosis, Micropinocytosis, Proteomics

## Abstract

**Background:**

Tumor cells release extracellular vesicles (EVs) that contribute to the polarization of macrophages towards tumor-associated macrophages (TAMs). High expression levels of the RNA binding protein IGF2BP2/IMP2 are correlated with increased tumor cell proliferation, invasion, and poor prognosis in the clinic. However, there is a lack of understanding of whether IMP2 affects the cargo of cancer cell-derived EVs, thereby modulating macrophage polarization.

**Methods:**

EVs were isolated from IMP2-expressing HCT116 parental cells (WT) and CRISPR/Cas9 IMP2 knockout (KO) cells. EVs were characterized according to MISEV guidelines, microRNA cargo was assessed by microRNA-Seq, and the protein cargo was analyzed by proteomics. Primary human monocyte-derived macrophages (HMDMs) were polarized by EVs, and the expression of genes and surface markers was assessed using qPCR and flow cytometry, respectively. Morphological changes of macrophages, as well as the migratory potential of cancer cells, were assessed by the Incucyte^®^ system and macrophage matrix degradation potential by zymography. Changes in the metabolic activity of macrophages were quantified using a Seahorse^®^ analyzer. For in vivo studies, EVs were injected into the yolk sac of zebrafish larvae, and macrophages were isolated by fluorescence-activated cell sorting.

**Results:**

EVs from WT and KO cells had a similar size and concentration and were positive for 25 vesicle markers. The expression of tumor-promoting genes was higher in macrophages polarized with WT EVs than KO EVs, while the expression of *TNF* and *IL6* was reduced. A similar pattern was observed in macrophages from zebrafish larvae treated in vivo. WT EV-polarized macrophages showed a higher abundance of TAM-like surface markers, higher matrix degrading activity, as well as a higher promotion of cancer cell migration. MicroRNA-Seq revealed a significant difference in the microRNA composition of WT and KO EVs, particularly a high abundance of miR-181a-5p in WT EVs, which was absent in KO EVs. Inhibitors of macropinocytosis and phagocytosis antagonized the delivery of miR-181a-5p into macrophages and the downregulation of the miR-181a-5p target *DUSP6*. Proteomics data showed differences in protein cargo in KO *vs*. WT EVs, with the differentially abundant proteins mainly involved in metabolic pathways. WT EV-treated macrophages exhibited a higher basal oxygen consumption rate and a lower extracellular acidification rate than KO EV-treated cells.

**Conclusion:**

Our results show that IMP2 determines the cargo of EVs released by cancer cells, thereby modulating the EVs' actions on macrophages. Expression of IMP2 is linked to the secretion of EVs that polarize macrophages towards a tumor-promoting phenotype.

**Supplementary Information:**

The online version contains supplementary material available at 10.1186/s12964-024-01701-y.

## Background

Insulin-like growth factor 2 mRNA binding protein 2 (IGF2BP2/IMP2) belongs to the RNA-binding proteins highly expressed during fetal development and maturation in different tissues. In most adult tissues, however, this protein is either absent or expressed at low levels [1]. In several types of cancer, such as hepatocellular carcinoma (HCC) and colorectal cancer (CRC), IMP2 is overexpressed, which affects several tumor hallmarks, including cell proliferation, growth rate, invasion, as well as cell metabolism, and chemoresistance [2–6]. In addition to its effect on mRNA abundance, localization, and translation, IMP2 has also been shown to modify the abundance of cellular microRNAs (miRNAs) [7].

Macrophages are innate immune cells found in all tissues and play roles in development, homeostasis, and tissue repair [8]. Due to the high plasticity of macrophages, they can polarize towards different subsets and adopt diverse phenotypes, including M1 (classically activated macrophages) and M2 (alternatively activated macrophages) in response to various stimuli [9]. However, the actual polarization state of macrophages is more complex than the M1 or M2 classification, which is only used to define the extremes of macrophage functions [10, 11].

Abundant infiltration of macrophages occurs in the tumor microenvironment (TME), which is correlated with poor prognosis in patients [11]. Tumor-associated macrophages (TAMs) are the major immune cells in the TME, and their function and phenotype are influenced by several types of cells in the TME [12, 13]. We have previously demonstrated that the incubation of primary macrophages with tumor cell conditioned medium (TCM) induces a polarization state similar to TAMs [14]. This polarization state resembles an M2-like polarization but reflects more of a mixed phenotype.

Extracellular vesicles (EVs) are composed of phospholipid bilayers and are secreted by almost all cells [15]. EVs can interact with and deliver their diverse cargo to a variety of target cells. Therefore, they are important mediators of cell communication and can regulate biological processes in the recipient cells [16, 17]. EVs secreted from several types of tumor cells have been shown to polarize macrophages mainly towards an M2-like phenotype, which orchestrates angiogenesis, extracellular matrix remodeling, and tumor cell proliferation [18–21].

Due to the vital role of IMP2 in carcinogenesis and tumor progression, we hypothesized that IMP2 in cancer cells can determine the cargo of EVs released by cancer cells, thereby modulating the EVs' actions on macrophages. Therefore, in the present study, we have investigated the effects of EVs derived from IMP2-expressing parental and knockout (KO) cancer cells on primary human monocyte-derived macrophages and in vivo in zebrafish embryos.

## Methods

### Cell lines

HCT116 colorectal cancer cells (ATCC: CCL-247) and CRISPR-Cas9 IMP2 KO cells (clone 47–1) [6, 22] were grown in Dulbecco's Modified Eagle's Medium–High Glucose (DMEM, #D5796) supplemented with 10% fetal calf serum (FCS, #F7524), 2 mM L-glutamine (#G7513), 100 U/ml penicillin G, and 100 µg/ml streptomycin (#P4333). All the supplemented components were purchased from Sigma Aldrich (Germany). Cell line authentication was conducted by STR/DNA profiling. Cells were incubated at 37 °C and 5% CO_2_.

### Viability assay

HCT116 cells were seeded in a 6-well plate in complete DMEM (300,000 cells/well). The following day, cells were washed with PBS, after which fresh EV-depleted medium was added and incubated for 48 h at 37 °C. For sample preparation, cells were washed with PBS, detached with Accutase (#A6964, Sigma Aldrich, Germany), and centrifuged at 400 *g* for 4 min. The pellet was resuspended in 100 μl Annexin binding buffer with 5 μl FITC Annexin V (#640906, BioLegend, Germany) and 10 μl PI (#421301, BioLegend). After 15 min incubation in the dark, 400 μl binding buffer was added, and the samples were measured on a BD LSRFortessa (BD Biosciences). As a positive control, HCT116 cells were heated at 60 °C for 20 min. Data analysis was performed using FlowJo 10.8.1 (BD Biosciences).

#### Determination of protein concentration

The protein concentration was determined using the Pierce BCA protein assay kit (#23227, ThermoFisher Scientific, Germany) according to the manufacturer's instruction.

#### Isolation of cancer cell-derived EVs and EV-depleted medium

To prepare EV-deprived FCS, 30% FCS-containing DMEM was ultracentrifuged at 100,000 *g* for 18 h at 4 °C [23], followed by collecting half of the supernatant and filtering through a 0.2 μm stericup filter (Merck Millipore, Germany). The flow-through was used to prepare 10% EV-depleted medium. Cells were seeded in triple-layer flasks (#132913, ThermoFisher Scientific, Germany) until 70% confluency. After washing cells two times with PBS, 200 ml of the EV-depleted medium was added per flask and incubated for another 48 h. In the first approach for EV isolation, the ultracentrifugation (UC) method was applied. Briefly, 1000 ml of TCM was centrifuged at 300 *g* for 10 min at 4 °C, followed by 10,000 *g* for 30 min to remove cell debris and microvesicles, respectively. The resulting supernatant was ultracentrifuged at 100,000 *g* for 4 h at 4 °C using a Ti45 rotor (Beckman Coulter, USA). The pellet was washed with PBS and centrifuged at 100,000 *g* for 70 min. The collected EV pellet was resuspended in 100 μl PBS and stored at -80 °C. In the second approach, EVs were isolated using a Tangential Flow Filtration system (TFF). Briefly, 3000 ml of the TCM were collected. After sequential centrifugation, *ca.* 2700 ml were concentrated using a 370 cm^2^ 300 kDa cut-off fiber-modified polyethersulfone (mPES) membrane filter column (D06-E300-05-N, Repligen) operated on a KR2i TFF system (Repligen, USA). The EV-depleted fraction was collected on the permeate side. Concentrated EVs were subsequently washed with 1000 ml of 10 mM HEPES supplemented with 0.9% NaCl in the TFF system to remove impurities further. A final volume of 8 ml of the purified EVs was obtained on the retentate side and stored at − 80 °C. For proteomics analysis, the TFF EVs were further concentrated by ultracentrifugation at 100,000 *g* for 4 h at 4 °C using a Ti45 rotor (Beckman Coulter, USA); the pellet was resuspended in 150 μl PBS and stored at -80 °C.

### Characterization of EVs

#### Nanoparticle tracking analysis (NTA)

EV samples were diluted 100-fold in filtered 10 mM HEPES/0.9% NaCl and 1000 μl of the dilutions were injected into the sample chamber of a NanoSight LM10 (NanoSight Ltd). Three 30 s records were measured for each sample and analyzed by the NTA software to determine the concentration and size of the EVs.

#### Cryo-transmission electron microscopy (TEM)

A 3 µL droplet of the EV samples was placed on a holey carbon-covered TEM grid (Plano, type S147-4), blotted for 2 s, and then plunged into a bath of liquid ethane at -165 °C using a Gatan CP3 cryoplunger (Pleasanton). The frozen sample was transferred under liquid nitrogen to a Gatan cryo-TEM sample holder (model 914) and investigated at − 173 °C by low-dose brightfield TEM imaging (JEOL JEM-2100 LaB_6_). For image acquisition, a Gatan Orius SC1000 CCD camera was applied.

#### Western blot analysis (WB)

EV samples and cell lysate (100 µg) were denatured for 10 min at 95 °C in 4 × loading buffer (50 mM Tris–HCl, 1% SDS, 10% glycerol, and 0.004% bromophenol blue) and loaded on a 10% polyacrylamide-sodium dodecyl sulfate gel (SDS-PAGE) and then blotted on a PVDF membrane (#88518, ThermoFisher Scientific, Germany). After 1 h blocking in the blocking buffer (#MB-070, ROCKLAND, USA), samples were incubated overnight with the primary antibodies at 4 °C. Primary antibodies against CD9 (#MA1-80307, ThermoFisher Scientific) and CD63 (#sc-5275, SantaCruz, USA) were used in a 1:1,000 dilution in blocking buffer. The blots were washed three times with PBS-0.05% Tween 20 for 10 min and then incubated with IRDye 800 CW goat anti-mouse (LI-COR, 1:10,000) for 1 h at room temperature. Bound antibody was visualized with an Odyssey CLx imaging system at 800 nm (LICOR).

#### Proteomics

The samples were prepared from EVs, HCT116 parental, and KO cells in triplicates. The samples were lysed by adding 210 µL of 0.4% SDS in PBS and sonicated four times at an amplitude of 70% for 30 s (Bandelin Sonoplus). The samples were centrifuged, and the supernatant was used for the BCA assay (ThermoFisher Scientific). The proteome concentrations of the samples were adjusted to 100 µg/200 µL per sample. The proteome was then precipitated by adding 1 mL of ice-cold acetone and incubated overnight at -20 °C. The samples were centrifuged (16,900 *g*, 15 min, 4 °C) and the supernatant was discarded. The samples were washed twice with 1 mL of ice-cold methanol. For the protein digestion, the samples were suspended in 200 µL X-buffer (7 M urea, 2 M thiourea, 20 mM HEPES, pH = 7.5), followed by reduction of cysteine with 0.8 µL DTT (250 mM) (dithiothreitol) for 45 min at 25 °C, capping with 2 µL IAA (550 mM) (iodoacetic acid) for 30 min at 25 °C, addition of 3.2 µL DTT (250 mM), and incubation for another 30 min at 25 °C. For overnight digestion, 600 µL ammonium bicarbonate buffer (pH = 8.5) and trypsin (1 µg) (MS grade, Promega) were added. Digestion was stopped by adding 8 µL formic acid (FA). The peptide samples were desalted on SepPak C18 columns (Waters) according to the following protocol: Columns were primed by adding 1 mL of 100% and 80% acetonitrile (ACN) with 0.5% FA, followed by equilibration with 3 × 1 mL H2O + 0.1% trifluoroacetic acid (TFA), before samples were loaded and washed with 3 × 0.1% TFA. Samples were eluted in 2 mL LoBind vials (Eppendorf) by adding 3 × 250 µL 80% ACN + 0.5% FA, and dried with speedVac before being dissolved in 1% FA with a proteome concentration of 1 µg/µL. Samples were filtered (Merck Millipore) and transferred to QuanRecovery autosampler vials (Waters). Samples were analyzed using the nanoElute nano flow liquid chromatography system (Bruker, Germany) coupled to a timsTOF Pro (Bruker, Germany). Samples were loaded onto the trap column (Thermo Trap Cartridge 5 mm) and washed with 6 µL of 0.1% FA at a 10 µL/min flow rate. The peptides were then transferred to the analytical column (Aurora Ultimate CSI 25 cm × 75 µm ID, 1.6 µm FSC C18, IonOpticks) and eluted by gradient elution (eluent A: H2O + 0.1% FA, B: ACN + 0.1% FA; 0% to 3% in 1 min, 3% to 17% in 57 min, 17% to 25% in 21 min, 25% to 34% in 13 min, 34% to 85% in 1 min, 85% maintained for 8 min) at a flow rate of 400 nL/min. The captive spray nanoESI source (Bruker, Germany) was used to ionize the peptides at 1.5 kV and 180 °C dry temperature at a gas flow of 3 L/min. timsTOF Pro (Bruker, Germany) was operated in standard dia-PASEF long gradient mode with TIMS set to 1/K0, start at 0.6 Vs/cm2, end at 1.6 Vs/cm2 with a ramp and accumulation time of 100 ms each and a ramp rate of 9.43 Hz. The mass range was set from 100.0 Da to 1700 Da with positive ion polarity. The dia-PASEF mass range was set from 400.0 Da to 1201.0 Da with a mobility of 0.60 1/K0 to 1.43 1/K0 and a cycle time of 1.80 s. The collision energy for 0.60 1/K0 was set to 20.00 eV and for 1.6 1/K0 to 59.00 eV. The collision energy for 0.60 1/K0 was set to 20.00 eV and for 1.6 1/K0 to 59.00 eV. Tuning MIX ESI-TOF (Agilent) was used to calibrate m/z and mobility. Data were processed with DIA-NN (version 1.8.1), and proteins were identified using the reference proteome of Uniprot *Homo sapiens* (proteome ID: UP000005640, downloaded on 22/03/2023). Default settings were used, except that the precursor charge range was from 2 to 4, and C-carbamidomethylation was set as a fixed modification. "-relaxed-prot-inf" was added in the additional options to allow further data processing with the Perseus software (version 2.0.11.0). Missing values were set as 0, and differential protein abundance between different conditions was evaluated using a two-tailed Student's t-test with Benjamini–Hochberg correction (FDR < 0.05). The mass spectrometry proteomics data have been deposited to the ProteomeXchange Consortium *via* the PRIDE partner repository with the dataset identifier PXD052287.

#### Isolation of human monocyte-derived macrophages (HMDMs)

Buffy coats were obtained from healthy blood donors (Blood Donation Center, Saarbrücken, Germany), authorized by the local ethics committee (approval no. 173/18). Written informed consent was obtained from all participants (a total of 25 individual cell isolation). Peripheral blood mononuclear cells (PBMCs) were isolated by density gradient centrifugation using lymphocyte separation medium 1077 (#C-44010, PromoCell, Germany) in LeucoSep tubes (#227290, Greiner, Austria). PBMCs were sorted for CD14^+^ cells using CD14 magnetic beads (#130–050-201, Miltenyi, Germany). Monocytes were seeded at a density of 500,000 cells/well in a 12-well plate and differentiated in complete RPMI medium supplemented with 20 ng/ml human recombinant colony-stimulating factor (M-CSF, Miltenyi, #130–096-492) for 6 days with changing the medium on day 4. Cells were then polarized for 24 h with EVs isolated with the UC method at a cell:EV ratio of 1:10,000 and the TFF system (1:10,000 and 1:20,000). TAM-like macrophages were generated after the cultivation of macrophages in TCM derived from HCT116 supplemented with 20 ng/ml M-CSF for 48 h [14].

#### Internalization assay

10^10^ EVs in 10 mM HEPES supplemented with 0.9% NaCl were incubated with 50 µM carboxyfluorescein succinimidyl ester (CFSE, #423801, BioLegend) for 2 h at 37 °C. The unlabeled dye was removed by an Amicon filter (#UFC501008, Sigma-Aldrich). EVs were then incubated with primary macrophages for 24 h in SFM 1 × medium (1:10,000 cell:EV ratio). Cells were fixed with 2% paraformaldehyde (PFA) for 20 min, followed by Hoechst 33342 staining for 5 min (12 µg/ml, #62249, ThermoFisher Scientific). The images were obtained with an LSM 710 NLO confocal microscope (Zeiss, Germany).

#### Macrophage morphology analysis

HMDMs were isolated, differentiated, and polarized with EVs or TCM as described above. Cells were then imaged with an Incucyte^®^ S3 system, and the analysis of cell morphology was performed with the Incucyte^®^ cell-by-cell analysis software and grouped in a round or elongated phenotype based on their eccentricity [24].

#### RNA Isolation, reverse transcription and qPCR

RNA was isolated using the High Pure RNA isolation kit (#11828665001, Roche Diagnostics) as recommended by the manufacturer. An equal amount of RNA was reverse transcribed using the high-capacity cDNA reverse transcription kit (#4368813, ThermoFisher Scientific, Germany). cDNAs were analyzed by qPCR using 5xHOTFIREPol EvaGreen qPCR Mix (#08–24-00020, Solis BioDye, Estonia) in a CFX96 touch™ Real-Time PCR detection system (Bio-Rad, USA). All samples and standards were analyzed in triplicate, and data were normalized to the housekeeping gene *RNA18S5*. The sequence of the primers is listed in Table 1.

**Table 1:** Primer sequences used for qPCR

gene	Accession number	Forward (5´-3´)	Reverse (5´-3´)
*HIF1A*	NM_181054.3	CGGGGACCGATTCACCAT	TTTCGACGTTCAGAACTTATCTTTT
*ABCG1*	NM_016818.3	GCGCCAAACTCTTCGAGCTG	CGGATGCAACCTCCATGACAAA
*IL6*	NM_000600.5	ACATCCTCGACGGCATCTCA	TCACCAGGCAAGTCTCCTCATT
*MMP9*	NM_004994.2	TTCTGCCCGGACCAAGGATA	ACATAGGGTACATGAGCGCC
*VSIR*	NM_022153.2	CTACAAGCAAAGGCAGGCAG	TCCCTTGAATGTTGCTGTCCAT
*TNF*	NM_000594.4	CTCCACCCATGTGCTCCTCA	CTCTGGCAGGGGCTCTTGAT
*RNA18S5*	NR_003286.2	AGGTCTGTGATGCCCTTAGA	GAATGGGGTTCAACGGGTTA
*DUSP6*	NM_001946.4	GCAGCGACTGGAACGAGAAT	GAACTTACTGAAGCCACCTTCC
*IL1B*	NM_000576.2	GGCTGCTCTGGGATTCTCTT	AGTCATCCTCATTGCCACTGTAA
*tnf*	NM_212859.2	TACGGAGGCAAAAAGCCACT	AGAAGTGCTGTGGTCGTGTC
*il6*	NM_001261449.1	ATGACGGCATTTGAAGGGGT	TCAGGACGCTGTAGATTCGC
*eef1a*	NM_131263.1	AAGCCCATGTGTGTGGAGAG	CAACCTTTGGAACGGTGTGA

#### Flow cytometry analysis

The analysis was performed as described previously [14]. Briefly, polarized macrophages were washed with PBS and then detached with Accutase. After a brief centrifugation, cells were resuspended in FACS buffer (PBS, 2.5% FCS, 0.1% sodium azide), blocked in human Fc Block solution (#564220, BD Biosciences, USA) for 15 min on ice, and then stained with the antibodies for 30 min on ice. The antibodies used in this procedure included CD14-APC (#555399, BD Biosciences), CD163-PE-CF594 (#562670, BD Biosciences), CD80-BB515 (#565008, BD Biosciences), CD86-BV421 (clone BU63, BioLegend), CD206-Alexa Fluor (#32111, BioLegend), CD40-APC (#325804, BioLegend), HLA-DR-PerCP-Cy5.5 (#560652, BD Biosciences), APC Mouse IgG1, κ (#554681, BD Biosciences), PE-CF594 Mouse IgG1, κ (#562292, BD Biosciences), BB515 Mouse IgG1, κ (#564416, BD Biosciences), PerCP-Cy5.5 Mouse IgG2a, κ (#552577, BD Biosciences), BV421 Mouse IgG2b (#400341, BioLegend), Alexa Fluor IgG1, κ (#400129, BioLegend), and APC Mouse IgG1, κ (#400122, BioLegend). Cells were washed three times with the buffer and then resuspended in 1% paraformaldehyde in PBS before analysis. The median fluorescence intensity of the singlet cells was used to quantify surface marker expression.

#### Enzyme‑linked immunosorbent assay (ELISA)

The secretion of tumor necrosis factor (TNF) and interleukin 6 (IL6) was measured in macrophage supernatants by using ELISA MAX™ Deluxe Set for TNF (#430204) and IL6 (#430504) as recommended by the manufacturer (BioLegend).

#### Gelatin zymography assay

The assay was performed based on a previously described procedure [25]. Briefly, polarized macrophage supernatant was loaded onto a 10% SDS-PAGE gel containing 1 mg/ml gelatin type A (#9000–70-8, Sigma Aldrich, Germany). SDS was removed from the gel by washing in 2.5% TritonX-100 for 1 h. The enzymatic reaction occurred by overnight incubation of the gel at 37 °C in the reaction buffer (50 mM Tris, pH 7.5, 200  mM NaCl, 5 mM CaCl_2_, and 0.02% Brij-35). The gel was then stained with Coomassie Blue solution (#20278, ThermoFisher Scientific, Germany) for 30 min and then shortly destained in 10% MeOH and 5% acetic acid. The area of gelatin degradation identified as MMP9 activity was visualized by an Odyssey CLx imaging system.

#### Migration assay

The assay was performed on a monolayer of HCT116 cells in an ImageLock 96 well plate (40,000 cells/well) as described previously with some modifications [24]. Primary macrophages were differentiated and polarized with EVs or TCM for 24 h in macrophage SFM 1 × medium (SFM, #12065074, ThermoFisher Scientific, Germany). The scratch was made by a wound maker (Sartorius, Germany), followed by the addition of primary macrophage supernatant. The images were captured every 4 h by an Incucyte® S3 system. The percentage of wound confluency was quantified using the Incucyte^®^ migration software.

#### Small RNA library preparation

The library was prepared from EVs, HCT116 parental and KO cells, each in quadruplicate. RNAs from EVs and cells were isolated using miRNeasy Serum/Plasma kit (#217184, Qiagen) and the miRNeasy Mini kit (#217004, Qiagen), respectively, according to the manufacturers' protocols. The RNA concentration was quantified by a Nanodrop spectrometer (ThermoFisher Scientific, USA) at 260 nm. The small RNA library was prepared according to the MGIEasy small RNA library preparation kit (#1000005269, China). Briefly, after 3' and 5' adapter ligation, cDNA was synthesized. After PCR amplification, the product was loaded onto a 6% TBE PAGE gel, and the portion of the gel corresponding to the appropriate size (100–120 bp) was extracted. The purified PCR product was subjected to denaturation and single-strand circularization, followed by enzymatic digestion and cleanup. The purified product was quantified with a Qubit ssDNA assay kit (#Q10212, Invitrogen). The resulting small RNA libraries were sequenced by MGI Tech (China).

#### Computational analysis of miRNA-Seq data

Fastq sequencing files were analyzed using miRMaster's pipeline with default parameters as previously described [26] and using miRbase as a reference (release 22.1) [27]. As an output, miRMaster generated a list with the expression of all mapped miRNAs. Expression was normalized to reads per million of miRNA mapped reads. Differentially expressed miRNAs were calculated on normalized values using a two-tailed Student's *t*-test and adjusted for multiple testing using Benjamini-Hochberg. Principal Component Analysis (PCA) was performed on the miRNA expression data using the prcomp function from R. miRNA targets were obtained using miRTargetLink 2.0 and filtering targets supported by strong evidence [28]. Enrichment analysis of KEGG pathways was performed using miEAA [29]. The raw and processed data were deposited in the Gene Expression Omnibus repository (GSE235115).

#### qPCR with miRNAs

RNA (10 ng) was used as the starting material for reverse transcription (miRCURY LNA RT kit, # 339340, Qiagen, Germany). With a 1:60 dilution of the cDNA, qPCR was performed using miRCURY LNA SYBR Green PCR kit (#339346, Qiagen) as recommended by the supplier. miRNA PCR assays were purchased from Qiagen for miR-181a-5p, miR-181a-3p, and miR-452-5p, as well as two reference miRNAs (U6 and 16-5p). Data were normalized to the reference genes, and fold change was calculated using the 2^^ − (∆∆Ct)^ method.

#### miRNA mimic transfection

The mirVana miRNA mimics for miR-181a-5p (#MC10421), miR-181a-3p (#MC10381), miR-452-5p (#MC12509), and the non-target control #1 (NTC, #4464058) were purchased from ThermoFisher Scientific. Non-polarized macrophages were transfected in a 12-well plate (250,000 cells/well) with the miRNA mimic and NTC at a final concentration of 100 nM, using 3 µl Lipofectamine 3000 (#L3000008, Invitrogen) in SFM 1 × medium without antibiotics. RNAs were isolated 24 h after transfection and dual specificity phosphatase 6 (*DUSP6)* gene expression was assessed by qPCR.

#### EV uptake inhibition

Primary macrophages were pre-treated with either 100 µM of LY294002 (#L9908), 80 µM of Dynasore (#D7693), or 200 µM of 5-(N-Ethyl-N-isopropyl)-Amiloride (EIPA, #A3085, Merck Millipore, Germany) for 1 h at 37 °C. Subsequently, CFSE-labeled EVs were added, and cells were incubated for an additional 6 h. Afterward, samples were prepared for flow cytometry analysis. To investigate whether the inhibition of uptake leads to a reduction in miRNA delivery and subsequent changes in gene expression, macrophages were pre-treated with EIPA or LY294002 (LY) for 1 h and then incubated with WT and KO EVs for 6 h (1:20,000 cell:EV ratio). Afterwards, RNA was isolated and quantification of miR-181a-5p was performed by qPCR. Additionally, changes in the gene expression of *MMP9, HIF1A*, and *VSIR* were assessed.

#### Treatment of macrophages with boiled EVs

WT and KO EVs were boiled at 95 °C for 5 min. Subsequently, boiled and non-boiled EVs were incubated with macrophages for 24 h (1:20,000 cell:EV ratio). Following this incubation, RNA was isolated, and the levels of miR-181a-5p were quantified by qPCR. In addition to miRNA quantification, changes in *MMP9*, *HIF1A*, and *VSIR* gene expression were assessed.

### Metabolic activity of primary macrophages

#### MTT assay

The metabolic activity of primary macrophages was assessed using an MTT assay. Forty thousand cells were seeded in a 96-well plate in complete RPMI medium. The day after seeding, EVs isolated with the TFF system (1:20,000–1:1,250 cell:EV ratios) or the UC method (1:20,000–1:625) were added to the cells in complete medium. 24 h after treatment, cells were incubated for 1 h with 3-(4,5-dimethylthiazol-2-yl)-2,5-diphenyltetrazolium bromide (MTT solution, #M5655, Sigma Aldrich, USA), followed by the addition of DMSO to dissolve the violet formazan crystals. Absorbance was measured at 560 nm in a microplate reader (GloMax^®^, Promega, USA). Metabolic activity was expressed as a percentage compared to M0. To determine changes in the number of cells upon treatment with EVs, cells were counted with Incucyte cell-by-cell analysis software.

#### Mito stress assay

Mito stress tests were performed using an Agilent Seahorse 96XF instrument and a respective kit as recommended in the manufacturer's protocol (#103015–100). In brief, 110,000 differentiated macrophages were seeded into a 96-well Seahorse XF Cell Culture Microplate and then polarized with EVs at different cell:EV ratios (1:10,000 and 1:20,000) for 24 h before measurement. Macrophages were then incubated with Seahorse RPMI medium for 1 h at 37 °C followed by treatment with 1 µM oligomycin, 2 µM carbonyl cyanide-*p*-trifluoromethoxy-phenylhydrazone (FCCP), and 0.5 µM rotenone/antimycin A. After the measurement, cells were stained with 12 μg/ml of Hoechst dye (#62249, ThermoFisher Scientific, Germany), and the fluorescence intensity was quantified in a plate reader (Cytation5, BioTek), which was used for normalization to ensure equal cell distribution among the wells. Data were analyzed by the Seahorse Wave Software (Agilent Technologies), and the oxygen consumption rate (OCR) and the extracellular acidification rate (ECAR) were calculated.

### In vivo zebrafish embryo model

#### Zebrafish husbandry

Zebrafish husbandry and all experiments were performed in accordance with the European union directive on the protection of animals used for the scientific purpose (Directive 2010/63/EU) and the German Animal Welfare Act (§11 Abs. 1 TierSchG) and maintained using standard methods [30]. Zebrafish were kept in an automated aquatic eco-system (PENTAIR, Apopka, UK) and monitored regularly to maintain the following parameters: pH = 7.0 ± 0.1, temperature = 28 ± 0.5 °C, conductivity = 800 ± 50 μS, and light–dark cycle = 14 h–10 h. Transgenic zebrafish line Tg(mpeg1.1:GFP)ka101 with green fluorescent embryonic macrophages was used in this study. Embryos and larvae were maintained in fresh 0.3 × Danieau's (17 mM NaCl, 2 mM KCl, 0.12 mM MgSO_4_, 1.8 mM Ca(NO_3_)_2_, 1.5 mM HEPES, 1.2 µM methylene blue, pH = 7.1) at 28 °C. At a maximum of 120 h post-fertilization (hpf) larvae were euthanized by submersion in ice water for at least 12 h.

#### Maximum tolerated concentration (MTC)

To determine the MTC of EVs, 20 zebrafish larvae per condition were injected with WT EVs, KO EVs, or HEPES buffer (4 nl, equal to 7,500 EVs) at 3 days post fertilization (dpf) into the yolk sac. 8, 24, and 48 h post-injection (hpi) the number of live larvae was counted and plotted in a Kaplan–Meier curve.

#### Injection of EVs into yolk sac and sample preparation

Embryos were kept at 28 °C in 0.3 × Danieau's solution with 0.003% propylthiouracil (PTU). At 3 dpf, embryos were dechorionized with 1 mg/ml pronase, anesthetized by immersion in 250 µg/ml tricaine (3-amino benzoic acid ethyl ester, #886–86-2 Sigma Aldrich, Germany). Zebrafish larvae were injected either with 4 nl of WT EVs, KO EVs, or HEPES buffer (n = 3, 100–150 larvae/condition, 7,500 EVs/larva) into the yolk sac using a FemtoJet microinjector (Eppendorf, Germany). Larvae were anesthetized by immersion in 250 µg/ml tricaine 18 h after injection. Sample preparation for macrophage isolation was performed as described previously with some modifications [31]. The larvae were homogenized using a 70 μm cell strainer with a syringe plunger, washed with cold working buffer (PBS, 2 mM EDTA, 2% FCS), and centrifuged at 400 *g* for 5 min at 4 °C. The pellet was suspended in the buffer, filtered through a 40 μm cell strainer, washed, and centrifuged again at 400 *g* for 5 min at 4 °C. The pellet was resuspended in 200 μl buffer and kept on ice until FACS analysis.

#### Fluorescence-activated cell sorting (FACS) analysis

Forward scatter (FSC) and side scatter (SSC) were used to determine the cells and exclude cell debris. As initial tests revealed no doublet formation, cells were directly subjected to fluorescence gating. Samples from wildtype zebrafish larvae (zebrafish without GFP^+^ macrophages, AB line) were used to exclude cells with autofluorescence and set the initial gate. Cells were sorted by endogenous eGFP expression with excitation at 488 nm, and emission was detected in FL2 (525/50 nm) using the SH800S cell sorter (Sony, USA).

#### RNA Isolation, reverse transcription and qPCR

RNAs derived from zebrafish macrophages were extracted by RNeasy micro kit (#74004, Qiagen) following the manufacturer's instructions. cDNA and qPCR experiments were performed as described for HMDMs. The primers are listed in Table 1, and qPCR data were normalized to the housekeeping gene *eef1a*.

#### Statistical analysis

GraphPad Prism 9 software (GraphPad, USA) was used for data analysis. The means of two groups were compared with Student's *t*-test, and for more than two groups, a one-way ANOVA test was applied, followed by Bonferroni's post-hoc test. Figures are plotted with individual values from each donor, represented by a different color. All data are presented as mean ± SD, and *p* < 0.05 was considered statistically significant. *p* < 0.05 (*), *p* < 0.01 (**), *p* < 0.001 (***), *p* < 0.0001 (****). *p* < 0.05 (#), *p* < 0.01 (##), *p* < 0.001 (###), *p* < 0.0001 (####).

## Results

### Isolation and characterization of cancer cell-derived EVs

**Fig. 1:**
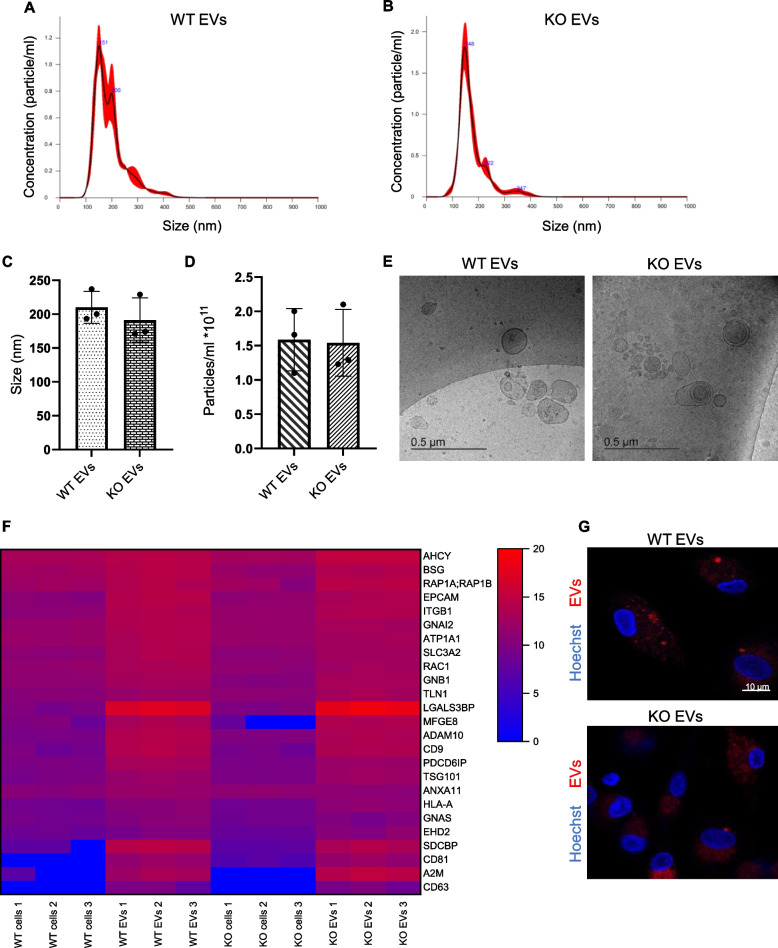
Characterization of HCT116 parental and KO cell-derived EVs isolated *via* the TFF system. **A**,** B** Representative NTA size distribution profiles of isolated EVs. **C**,** D** NanoSight quantification of EV preparations' size (C) and concentration (D) are shown as mean ± SD, n = 3 individual EV isolation, triplicates).** E** Cryo-TEM images of EVs, scale bar: 500 nm.** F** Abundance of 25 vesicle markers in WT and KO cells and EVs. Values are shown as log_2_ signal intensity of three independent preparations. **G** Cellular internalization of EVs into primary macrophages (1:10,000 cell:EV). Cells were incubated for 24 h with WT and KO EVs labeled with CFSE. Magnification, 63x, scale bar: 10 µm

Cancer cells were cultured in EV-depleted medium for 48 h, and TCM was collected for EV isolation. Annexin V/PI staining verified that the EV-depleted medium did not induce apoptosis or necrosis (Fig. S1). EVs isolated from the TCM of HCT116 parental and KO cells were initially obtained by the UC method, but due to the necessity of large numbers of EVs, we switched to the TFF system. Accordingly, we repeated most of the experiments using EVs isolated with the TFF system during our research. EVs isolated by both methods were thoroughly characterized according to MISEV guidelines [32]. The size and concentration of the purified EVs were determined by NanoSight nanoparticle tracking analysis (NTA) (Fig. 1A, B and Fig. S2A, B). NTA results showed that all EV samples fell within the expected size of around 200 nm in diameter with a concentration of *ca.* 10^11^ particles/ml (Fig. 1C, D and Fig. S2C, D). Visualization by cryo-TEM confirmed the spherical morphology of EVs with a size of around 200 nm (Fig. 1E and Fig. S2E). The purified EVs were further analyzed for EV markers by Western blotting and a proteomics approach. CD9 and CD63 were abundantly present in the EVs isolated with the UC method (Fig. S2F). For EV samples obtained with the TFF system, proteomics data showed that WT and KO EVs are positive for several vesicle markers [33]. Figure 1F shows 25 vesicle markers with the highest enrichment in WT EVs compared to WT cells. KO EVs did not differ from WT-EVs in terms of marker abundance. To determine whether EVs are taken up by primary macrophages, EVs were fluorescently labeled with CFSE and added to the cells. CLSM microscopy showed that EVs from both cell types were taken up by macrophages (Fig. 1G).

### EVs alter the macrophage phenotype

**Fig. 2:**
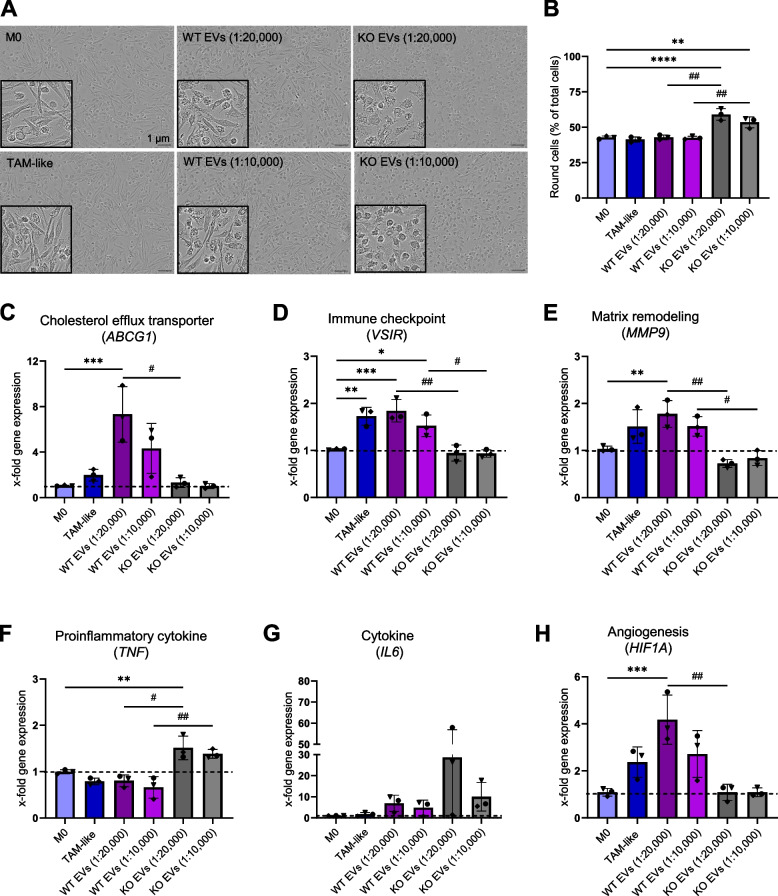

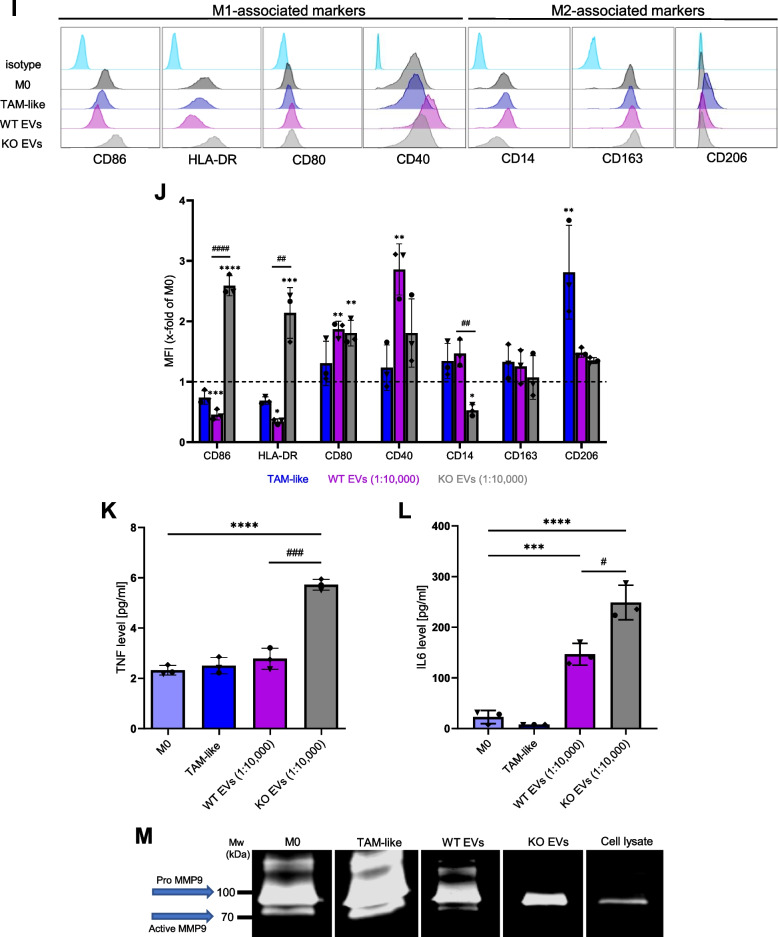
EVs change cell morphology, gene expression levels, surface markers, secretion of cytokines, and MMPs **A**,** B** Cells were grouped based on their eccentricity into a round or elongated phenotype by the Incucyte^®^ cell-by-cell analysis software. **A** Representative images of macrophages, either non-polarized (M0), polarized for 24 h with TCM (TAM-like) or with EVs (1:20,000 and 1:10,000) that were isolated with the TFF system. **B** Respective quantification of the cell population with round morphology (n = 3 individual donors, triplicates). **C-H** Gene expression in M0 and macrophages polarized with either EVs or TCM was assessed by qPCR after 24 h incubation (n = 3 individual donors, triplicates). **I**,** J** Surface marker expression was quantified in M0 and polarized macrophages by flow cytometry. Primary macrophages were incubated with EVs isolated with the UC method at a ratio of 1:10,000 (cell:EV), and TCM for 24 h. **I** Representative histograms. **J** Median fluorescence intensities (MFIs) are shown as x-fold of M0 (n = 3 individual donors, duplicates). **K-L** Macrophages were polarized for 24 h with TCM and EVs at a ratio of 1:10,000 (cell:EV) that were isolated with the UC method. The secretion of TNF (K) and IL6 (L) was quantified in M0 and EV-polarized macrophages by ELISA (n = 2 individual donors, triplicates). Data are represented as mean ± SD, and *p* < 0.05 is considered significant. * indicates a significant difference between treatments and M0. # shows a significant difference between WT and KO EVs. **M** Comparison of MMP9 secretion into macrophage supernatant by gelatin zymography assay (n = 2 individual donors). HCT116 cell lysate was used as a positive control

Macrophage morphology is linked to their polarization state, i.e., elongated cells exhibit a more M2-like phenotype [34]. Potential changes in the macrophage phenotype upon uptake of EVs from either WT or KO cells were assessed by an Incucyte^®^ live cell analysis system. Our results showed that when differentiated primary human macrophages were polarized with KO EVs, the percentage of round cells was significantly higher compared to the WT EV-treated and TAM-like macrophages (Fig. 2A, B). This indicates that KO EVs skew the macrophages towards an M1-like phenotype. A similar pattern was observed in macrophages that were polarized with UC-derived EVs for 24 h (Fig. S3).

### EVs modulate macrophage gene expression

Changes in the expression of genes characteristic for macrophage polarization and the immune regulatory receptor VISTA (gene name *VSIR*) were assessed by qPCR (Fig. 2C-H and Fig. S4) [14, 35]. We found that incubation of macrophages with WT EVs isolated with the TFF system and TCM resulted in a higher expression of the cholesterol efflux transporter G1 (*ABCG1*), V-set immunoregulatory receptor (*VSIR*), matrix metalloproteinase (*MMP9*), and hypoxia-inducible factor 1-alpha (*HIF1A*) compared to the non-polarized M0 and KO EV-polarized macrophages. *IL6* was upregulated in all subsets of macrophages, and the highest level of upregulation was observed in KO EV-polarized cells. Furthermore, the expression of the proinflammatory cytokine *TNF* in the KO EV-treated macrophages was higher than in WT- and TCM-incubated macrophages (Fig. 2F). The expression of *IL1B* was also upregulated in macrophages polarized with KO EVs (1:10,000, cell:EV ratio) (Fig. S4E). A similar pattern in gene expression was observed with EVs obtained with the UC method (Fig. S4A-D).

### Cell surface markers change in polarized macrophages

Changes in M1- and M2-associated surface markers were investigated by flow cytometry (Fig. 2I, J). The results revealed that M1-associated CD86 and HLA-DR were more abundant in the KO EV-treated macrophages compared to other treatments and non-polarized M0, while the abundance of CD14, linked to an M2-type polarization, was decreased. On the other hand, TAM-like and WT EV-treated cells showed a lower expression of CD86 and HLA-DR and a higher expression of CD14. A higher expression of CD80 was observed in both EV-treated groups. The M1-associated surface marker CD40 exhibited higher levels in WT EV-treated macrophages compared to M0. CD206, an M2-associated marker, was most abundant in macrophages polarized towards a TAM-like phenotype. The expression of CD163, also associated with an M2-like polarization, did not show significant changes in TAM-like or EV-treated macrophages. Overall, these results indicate that EVs, similar to TCM, can induce a mixed M1- and M2-like phenotype [14].

### TNF and IL6 secretion and MMP9 activity in macrophage supernatant

We quantified the secretion of the proinflammatory cytokines TNF and IL6 into the macrophage supernatant with ELISA 24 h after polarization (Fig. 2K, L). TNF secretion by macrophages polarized with WT EVs and TCM (TAM-like) remained unchanged, while the secretion of TNF was increased in the cells that were polarized with KO EVs (Fig. 2K). IL6 was secreted by both WT- and KO-polarized macrophages, but the IL6 levels in KO EV-treated macrophage supernatant were significantly higher. In TAM-like macrophages, the secretion of IL6 was slightly lower than M0 (Fig. 2L). To assess the secretion of MMP9, gelatinase activity was measured. The result of the zymography assay showed a significantly higher MMP9 activity in TAM-like supernatants compared to the other treatments and M0. Moreover, WT EV-polarized macrophage supernatant displayed a higher MMP9 activity than the supernatant of KO EV-treated cells (Fig. 2M).

### WT EV-polarized macrophage supernatant increases cancer cell migration

**Fig. 3:**
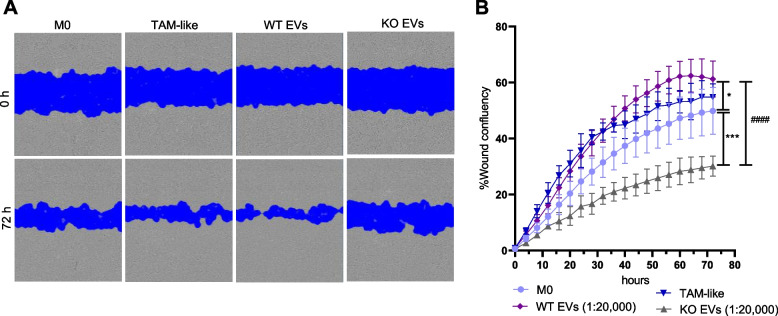
WT EV-polarized macrophage supernatant increases cancer cell migration. Primary macrophages were polarized with EVs isolated with the TFF system at a ratio of 1:20,000 (cell:EV), and TCM (TAM-like) for 24 h. The supernatant from the non-polarized M0 and polarized macrophages was added to the HCT116 cells, and the wound closure was assessed over 72 h with an Incucyte live cell imaging system. **A** Representative images of cancer cells at times 0 and 72 h after the addition of the supernatants. **B** Statistical analysis was performed for the last time point using one-way ANOVA followed by Bonferroni's post-hoc test. Data are presented as mean ± SD, and *p* < 0.05 was considered statistically significant (n = 3 individual donors, triplicate). * indicates a significant difference between treatments and M0. # shows a significant difference between WT and KO EVs

To test whether polarized macrophage supernatant affects cancer cell migration, we evaluated the migration capacity of cancer cells in a scratch wound assay. We observed that WT EV-polarized macrophage and TAM-like supernatants were able to significantly increase cancer cell migration compared to the KO EV-polarized macrophage supernatant (Fig. 3A, B).

### Macrophages derived from WT EV-injected zebrafish larvae show lower expression of *tnf *and *il6*

**Fig. 4:**
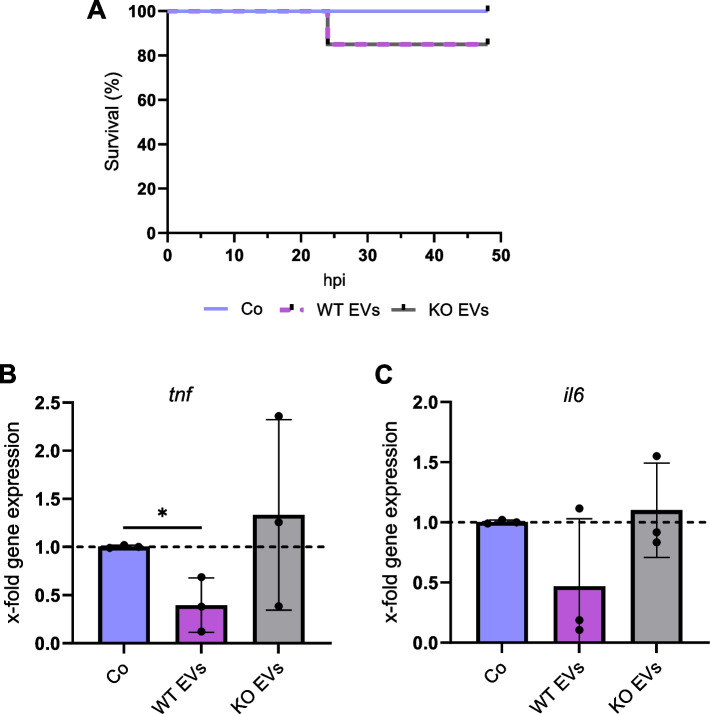
Zebrafish embryo model. mpeg1-eGFP transgenic zebrafish larvae were injected with 7,500 EV/larva and 18 h after injection, eGFP^+^ macrophages were isolated using FACS. **A** Kaplan–Meier graph shows the percentage of survival up to 48 h post-injection with EVs isolated with the TFF system. **B**,** C** Changes in the expression of *tnf* and *il6* in macrophages were quantified by qPCR, and data were normalized to the housekeeping gene *eef1a*. Statistical analysis was performed using one-way ANOVA followed by Bonferroni's post-hoc test. * shows a significant difference between Co and EVs. Data are presented as mean ± SD, and *p* < 0.05 was considered statistically significant (n = 3 biological replicates, 100–150 embryos/condition). Co: larvae injected with HEPES buffer, hpi: hours post-injection

Our data show a substantially different potential of EVs to polarize primary human macrophages depending on whether cancer cells express IMP2. We aimed to confirm these effects in an emerging 3R compatible in vivo model using zebrafish embryos. Toxicity tests revealed 3 out of 20 (15%) larvae died 24 h after the injection with either WT or KO EVs. Still, the treatments did not induce further toxicity over the next 24 h (Fig. 4A). Injection of HEPES buffer did not affect zebrafish larvae viability. FACS analysis resulted in between 0.35%-0.55% fluorescent cells corresponding to the macrophages (Fig. S5). In the following step, changes in the gene expression of proinflammatory cytokines *tnf* and *il6* were investigated by qPCR. Our data showed that the levels of both cytokines were reduced after the injection of WT EVs compared to the KO EV- and buffer-injected larvae (Fig. 4B, C).

### WT and KO EVs contain different miRNA cargo

**Fig. 5:**
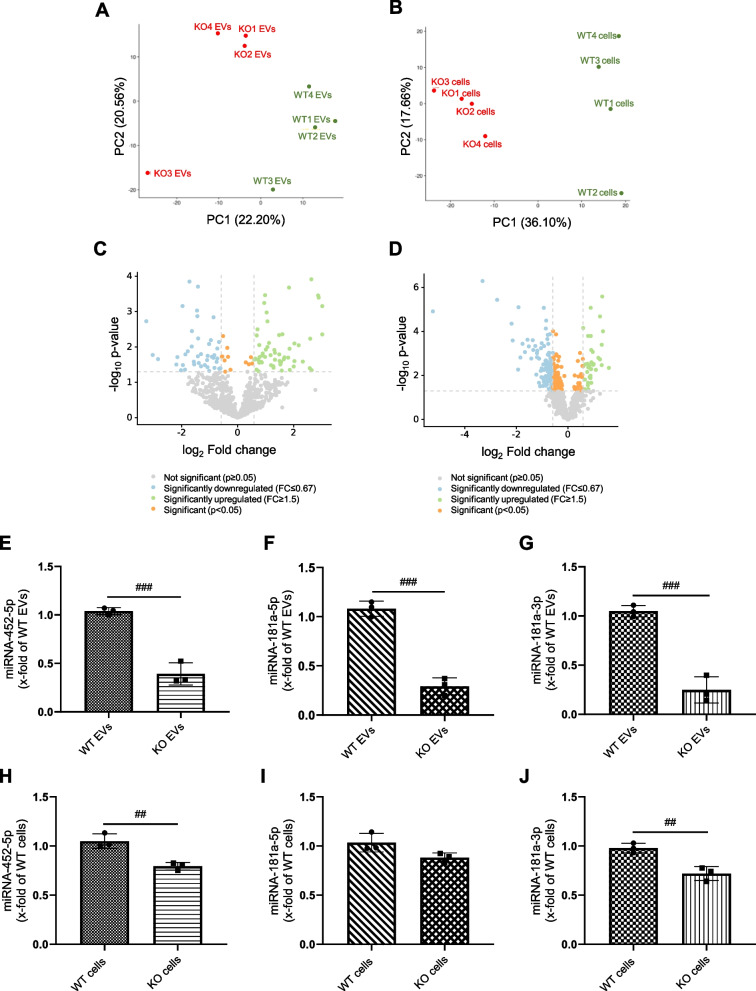

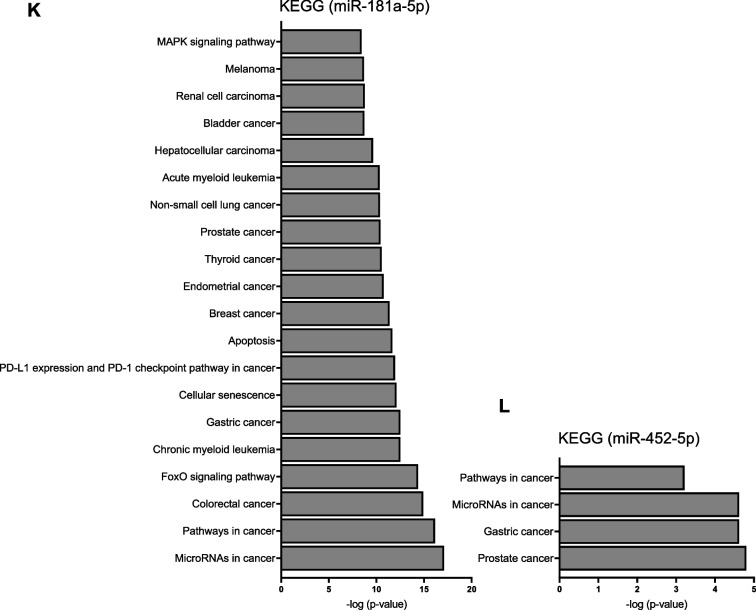
miRNA-Seq data. **A**,** B** PCA shows a clear distinction between WT and KO EVs (left) and HCT116 parental and KO cells (right). **C**,** D** Differentially expressed miRNAs in EVs (right) and cells (left) are shown as volcano plots. Log_2_ Fold change is plotted against -log_10_ *p*-value (4 biological replicates). **E-J** The presence of miRNAs was assessed by qPCR in EV and cell samples. The content of miRNAs in EVs (E–G) and cells (H-J) was normalized to 16-5p and U6, respectively, and is shown as fold change relative to WT samples. Statistical analysis was performed using Student's *t*-test, and data are presented as mean ± SD, n = 3, triplicates. # shows a significant difference between WT and KO EVs. **K**,** L** KEGG pathway enrichment for miR-181a-5p and miR-452-5p. KEGG pathway enrichment for miR-181a-5p (left) and miR-452-5p (right) are shown. The analysis was performed using miRTargetLink 2.0

We sought to elucidate the mechanism of how IMP2 expression by cancer cells determines EV actions on macrophages. Since miRNAs represent essential mediators of cell–cell communication and IMP2 has been shown to affect the cellular abundance and action of miRNAs [7], we hypothesized that IMP2-expressing cells mediate at least some of their actions on macrophages *via* miRNAs. We, therefore, performed miRNA-Seq of both cells and EVs. Principal component analysis (PCA) showed a clear distinction between WT and KO EV replicates and among the HCT116 parental and KO cells (Fig. 5A, B). We observed a high correlation among the 4 biological replicates for each group (Fig. S6). Our cluster analysis of miRNA profiles showed that the expression of 51 miRNAs was altered in WT EVs compared to KO EVs (Supplemental Table S1). To confirm miRNA-Seq data, three miRNAs (miR-181a-5p, miR-181a-3p, and miR-452-5p) were selected, which showed a high fold change between WT and KO EVs and had higher abundance in WT EVs relative to KO EVs (Table 2). qPCR results verified the miRNA-Seq data, showing that miR-181a-5p, miR-181a-3p, and miR-452-5p are highly abundant in WT EVs compared to KO EVs (Fig. 5E-G). A similar pattern was observed between HCT116 parental and KO cells, although with less pronounced differences (Fig. 5H-J). In order to confirm that these alterations depend on the presence or absence of IMP2 and do not represent a clonal artifact, we assessed the expression of these three miRNAs in two other IMP2 KO clones, i.e., clones 47–2 and 47–6 [22]. Data for all three clones were very similar and confirmed the higher abundance of all three miRNAs in IMP2-expressing cells (Fig. S8).

**Table 2:** Selected differentially expressed miRNAs enriched in WT EVs

miRNA	log_2_FC (KO/WT)	RPM (read per million)
hsa-miR-181a-5p	-1.45	7330.93
hsa-miR-181a-3p	-1.55	45.14
hsa-miR-452-5p	-2.84	12.43

We then identified potential target genes that might be affected by these miRNAs delivered by EVs to macrophages. We used miRTarget Link 2.0 and focused on the strongly validated genes (Table 3). For miR-181a-5p, which was expressed at very high levels in WT EVs (Table 2), 74 validated target genes were identified, while the validated target genes for miR-181a-3p and miR-452-5p were only one and seven, respectively (Table 3).

**Table 3:** Validated targets of WT EV-enriched miRNAs based on miRTarget Link 2.0

hsa-miR-181a-5p			hsa-miR-181a-3p	hsa-miR-452-5p
NLK	XIAP	RUNX1	NANOG	BMI1
GATA6	GPR78	SAMHD1		DPYSL2
CDX2	RASSF6	NRAS		KRAS
PLAG1	PBX3	CEBPA		THRB
BCL2	MAP2K1	EGR1		LEF1
PROX1	DDX3X	TCF4		TCF4
KAT2B	KRAS	CTNNB1		CDKN1B
CDKN1B	NOTCH1	RASSF1		
ZNF763	TIMP1	INPP4B		
DDIT4	COL16A1	PRKN		
ATM	PGR	GPD1L		
HIPK2	TGFBRAP1	BAX		
BCL2L11	PPP3CA	PTEN		
HRAS	ATG5	PHLPP2		
RNF2	TGFBR1	TUSC3		
RALA	E2F5	CDKN1A		
SIRT1	PRKCD	MEG3		
PRAP1	RAP1B	CTDSPL		
DUSP6	PRKCD	TWIST1		
PTPN11	ABCG2	MAPK1		
DUSP5	WIF1	WIF1		
PTPN22	TERT	RGS16		
FOS	RGS5	MCL1		
MTMR3	IFNG	STAT3		
KLF6	AHR			

### EV uptake is inhibited by macropinocytosis and phagocytosis inhibitors

**Fig. 6:**
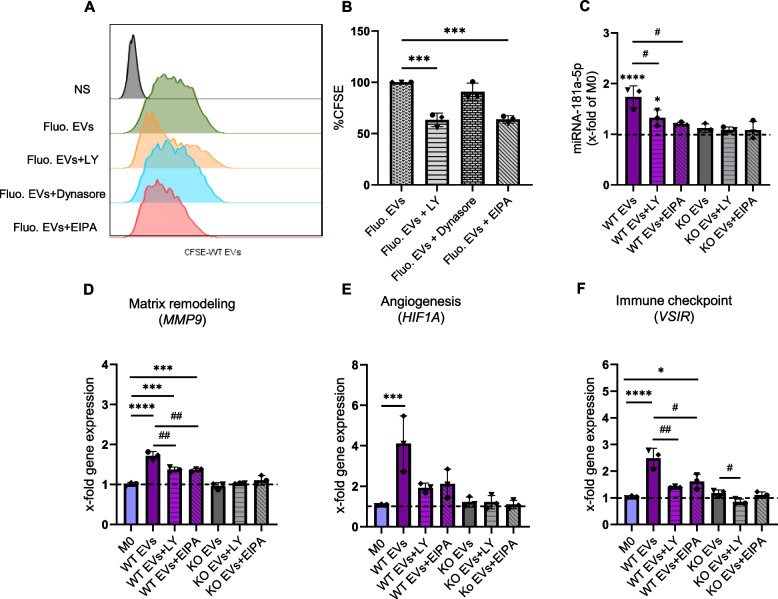
EV uptake is reduced with inhibitors. **A**,** B** Measurement of fluorescent EV uptake by flow cytometry. Macrophages were pre-treated with either LY294002 (LY), Dynasore, or EIPA for 1 h, followed by an additional 6 h incubation with CFSE-labeled WT EVs (n = 3 individual donors, one replicate). Representative histogram (A), Relative CFSE fluorescence compared to cells treated with fluorescent EVs but without inhibitor (B). NS: non-stained. **C** The amount of miR-181a-5p was quantified by qPCR after 1 h pre-treatment with the inhibitors and then 6 h incubation with WT and KO EVs (1:20,000 cell:EV ratio). **D-F** Changes in the expression of *MMP9*, *HIF1A*, and *VSIR* were assessed in macrophages incubated with EVs in the presence and absence of the inhibitors. Statistical analysis was performed using one-way ANOVA followed by Bonferroni's post-hoc test. Data are presented as mean ± SD, n = 3 individual donors, triplicates. * indicates a significant difference between treatments and M0. # shows a significant difference between EVs and EVs with inhibitors

To investigate the route(s) of EV uptake, we applied three commonly used inhibitors that have been reported to affect pathways of EV uptake in macrophages [36]. Since longer incubation times with the inhibitors impaired cell viability (data not shown), a treatment duration of 6 h was chosen. We observed that pre-treatment of macrophages with EIPA (macropinocytosis inhibitor) or LY294002 (phagocytosis inhibitor) led to an approximately 40% reduction in the uptake of fluorescently labeled EVs, while inhibition by Dynasore (clathrin-dependent endocytosis) was not statistically significant (Fig. 6A, B). To explore whether inhibiting EV uptake reduces the delivery of miR-181a-5p, the distinctly most abundant differentially expressed miRNA in WT EVs, and subsequent changes in gene expression, macrophages were incubated with EVs in the presence and absence of either EIPA or LY294002. Our findings revealed a significant reduction in the levels of miR-181a-5p in cells pre-treated with these inhibitors compared to cells treated with EVs alone (Fig. 6C). In addition, the inhibition of WT EV uptake resulted in lower expression of *MMP9*, *HIF1A,* and *VSIR*, three genes characterizing TAMs, compared to cells treated with WT EVs alone (Fig. 6D-F).

Next, we aimed to assess whether intact EVs are required to exhibit the action of the EV preparations we used. We observed that boiling abrogated the increase in miR-181a-5p expression in WT EV-treated macrophages (Fig. S9A). Furthermore, we assessed the expression of *MMP9*, *HIF1A*, and *VSIR* after treatment with boiled WT EVs, which resulted in no or minimal induction compared to non-boiled EVs (Fig. S9B-D).

### miR-181a-5p as a critical cargo in EVs released by IMP2-expressing cancer cells

**Fig. 7:**
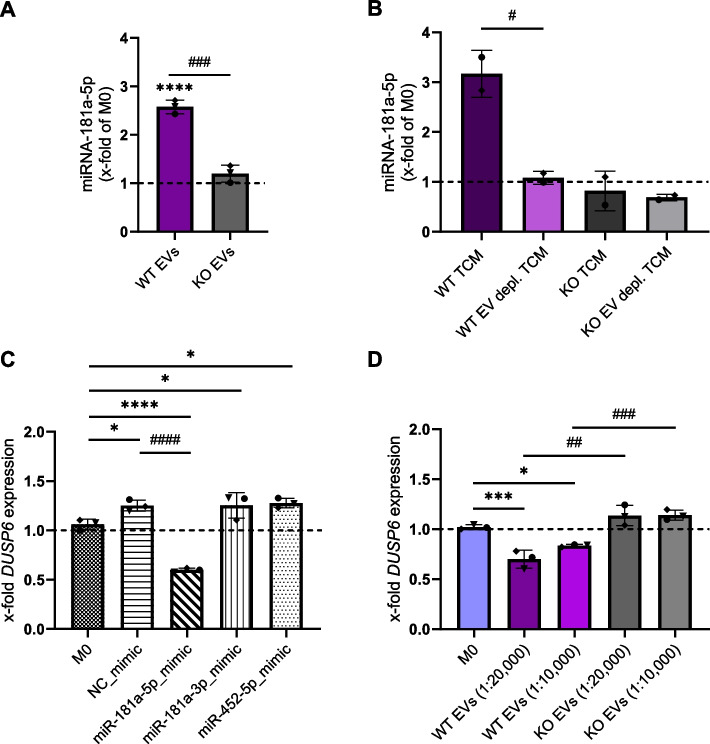
miR-181a-5p negatively regulates the expression of *DUSP6* MAPK phosphatase. **A** Content of miR-181a-5p in EV-treated macrophages. The amount of miR-181a-5p was quantified by qPCR after 24 h incubation with WT and KO EVs (1:20,000; cell:EV ratio). **B** Content of miR-181a-5p in macrophages polarized with WT and KO TCM and TFF EV-depleted TCM. The amount of miR-181a-5p was quantified by qPCR after 24 h incubation. **C** Non-polarized M0 were transfected with 100 nM miRNA mimics or NTC for 24 h, and the mRNA levels of *DUSP6* were assessed by qPCR. **D** mRNA levels of *DUSP6* in macrophages polarized with EVs. Statistical analysis was performed using one-way ANOVA followed by Bonferroni's post-hoc test. Data are presented as mean ± SD, n = 3 individual donors, triplicates. * indicates a significant difference between treatments and M0. # shows a significant difference between WT and KO EVs

KEGG pathway enrichment analysis showed much higher involvement of miR-181a-5p in several types of cancer and crucial signaling pathways compared to miR-452-5p and miR-181a-3p (Fig. 5K, L). One of the top pathways regulated by miR-181a-5p is the mitogen-activated protein kinase (MAPK) signaling pathway, which plays an important role not only in cancer [4, 5, 7], but also in the regulation of macrophage activation [37, 38]. DUSP6, a validated target of miR-181a-5p (Table 3), is a member of the MAPK phosphatase family, which negatively regulates MAPK members. Also, miR-452a-5p regulates the MAPK/DUSP6 pathway, which supports a tumor-suppressive macrophage phenotype [38, 39]. First, we quantified the amount of miR-181a-5p, which is highly upregulated in WT EVs compared to KO EVs, in macrophages treated with EVs. We observed a significantly higher amount of miR-181a-5p in WT EV-treated macrophages compared to KO EV-treated cells (Fig. 7A). Similarly, WT TCM increased miR-181a-5p expression, whereas KO TCM did not (Fig. 7B). To determine whether this WT TCM-induced increase was due to miRNA transfer *via* EVs, we incubated macrophages with EV-depleted TCM. EV depletion completely prevented the TCM-mediated increase in miR-181a-5p abundance (Fig. 7B). This finding conclusively shows that EVs from WT cells are responsible for the increased abundance of miRNA-181a-5p in WT TCM-treated macrophages. Furthermore, we could confirm *DUSP6* as a target gene of miR-181a-5p in HMDMs: the mRNA levels of *DUSP6* in macrophages transfected with the miR-181a-5p mimic were significantly decreased compared to the NTC-mimic or non-transfected M0 cells. In contrast, mimics for miR-452-5p or miR-181a-3p did not affect *DUSP6* mRNA levels (Fig. 7C). Moreover, the expression of *DUSP6* was decreased in macrophages polarized with WT EVs compared to M0 and KO EVs (Fig. 7D). On the other hand, KO EV-treated macrophages showed a slightly increased *DUSP6* expression compared to M0, but significantly higher than WT EV-polarized cells.

### Different protein composition of WT and KO EVs

**Fig. 8:**
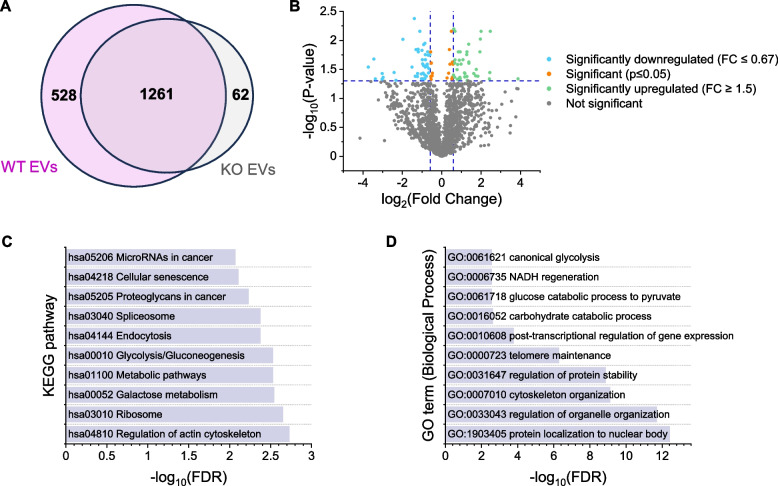
Proteomics data.** A** Total number of proteins detected in WT and KO EVs (= proteins with valid measurements in 3/3 preparations). **B** Differentially expressed proteins in KO *vs*. WT EVs. Log_2_ Fold Change is plotted against -log_10_ *p*-value. Proteins absent in one EV type are not shown. **C**,** D** KEGG Pathway (**C**) and GO term (**D**) enrichment for differentially abundant proteins

Besides miRNAs, proteins are an essential cargo involved in the effects of EVs in cancer progression [40]. Therefore, we analyzed the protein composition of WT and KO EVs by proteomics. In WT EVs, a total of 1789 proteins were detected in three out of three vesicle preparations. In KO EVs, the number was slightly lower at 1321, with most proteins detected in KO EVs overlapping with those in WT EVs (Fig. 8A). 149 proteins differed significantly in their abundance. Of these, 97 proteins were detected in WT and KO EVs, with 50 proteins showing a higher abundance in WT vesicles and 47 proteins being more highly abundant in KO vesicles (Fig. 8B). In addition, 50 significantly altered proteins, including IMP2, were exclusively present in WT EVs and two proteins in KO EVs (Supplemental Table S2). Interestingly, the protein exhibiting the largest difference in abundance between IMP2 WT and KO cells is CD44, i.e., a fragment of the hyaluronic acid receptor (Supplemental Table S2). With hyaluronic acid representing a major regulator of macrophage polarization [41], this finding might imply that the EV glycocalyx differs between these vesicles.

KEGG pathway analysis suggested that the differentially expressed proteins are involved in miRNA regulation, endocytosis, and regulation of the actin cytoskeleton. Moreover, proteins involved in several metabolic pathways, such as glycolysis, were significantly enriched. These included, for example, alpha-enolase, triosephosphate isomerase, pyruvate kinase, phosphoglucomutase-1, and hexokinase (Fig. 8C, Supplemental Table S2). The GO term analysis suggested an involvement of differentially abundant proteins in additional processes, e.g., in telomere maintenance and the organization of organelles, but also showed a clear link to metabolic processes (Fig. 8D, Supplemental Table S2).

### EVs change macrophage metabolism

**Fig. 9:**
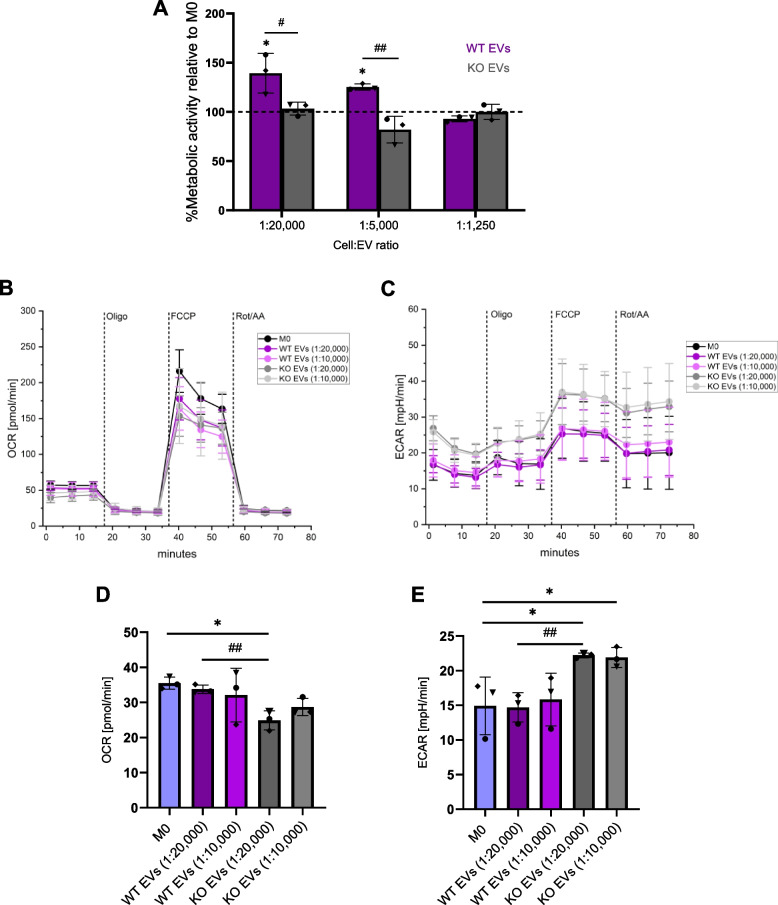
EVs affect macrophage metabolism. **A** The metabolic activity of primary macrophages was measured using an MTT assay 24 h after polarization with EVs isolated with the TFF system (1:20,000–1:1,250 cell:EV ratios). Data are presented as a percentage of metabolic activity relative to non-polarized M0 (mean ± SD, n = 3 individual donors, triplicates). **B-E** M0 macrophages were polarized with EVs isolated with the TFF system at different cell:EV ratios (1:20,000 and 1:10,000) for 24 h. OCR and ECAR were measured with a mito stress test using a Seahorse^®^ XFe96 Flux Analyzer. **B** Basal OCR. **C** ECAR in different macrophage subsets, and **D**,** E** Respective quantifications. Statistical analysis was performed using one-way ANOVA followed by Bonferroni's post-hoc test. Data are presented as mean ± SD (n = 3 individual donors, quintuplicate). * indicates a significant difference between treatments and M0. # shows a significant difference between WT and KO EVs

Macrophage phenotypes are linked to their metabolic profile. Proteomic analysis indicated that EV protein cargoes might influence metabolic processes. In addition, the miR-181a-5p target *DUSP6* has been proposed as a critical regulator of immune cell metabolism [42]. Therefore, we hypothesized that WT and KO EVs have different effects on macrophage metabolism. First, the changes in metabolic activity of macrophages polarized with EVs were examined using an MTT assay. While this assay is mainly used as a cytotoxicity assay, it is very sensitive toward metabolic alterations independent of cell number [24]. Cells were treated with different cell:EV ratios for 24 h. As shown in Fig. 9A and Fig. S10A, the polarization of macrophages with WT EVs resulted in a significantly higher metabolic activity compared to non-polarized M0 and the KO EV-polarized cells. Importantly, the number of cells after polarization with EVs remained unchanged (Fig. S10B). To further investigate changes in the bioenergetic profile of the polarized macrophages, mito stress tests were performed by a Seahorse^®^ analyzer in cells polarized with EVs for 24 h (Fig. 9B-E). At the basal level, KO EV-treated cells showed the lowest oxygen consumption rate (OCR, Fig. 9D). On the other hand, the extracellular acidification rate (ECAR) as an indirect measurement of glycolysis revealed the highest level in the KO EV-treated macrophages (Fig. 9E).

## Discussion

Even though the role of IMP2 has been investigated in tumorigenesis and cancer progression in different gastrointestinal cancers [4–6, 22], there is a lack of understanding of its effects on tumor-associated macrophages. Therefore, in the current study, we investigated the functional impact of EVs secreted from IMP2-expressing HCT116 and CRISPR/Cas9 IMP2 KO cells in vitro on primary human monocyte-derived macrophages and on macrophages isolated from zebrafish embryos after in vivo treatment with EVs.

We have previously shown that the cultivation of HMDMs with lung cancer TCM is a representative in vitro model for ex vivo TAMs [14]. In this study, we observed that TCM from HCT116 can polarize macrophages towards TAM-like cells, which are known as drivers of diverse hallmarks of cancer. The TAM-like macrophages induced by HCT116 TCM changed expression levels of TAM-associated genes and surface markers and their secretion of MMPs and cytokines. They also increased the migratory capacity of cancer cells, paralleled by morphological alterations. While multiple characteristics of TAM-like macrophages show similarity to an M2-like phenotype, the cells do neither exhibit a clear M1 nor M2 polarization. In fact, the heterogeneity of TAM phenotypes in the TME is well established, while the terms M1 and M2 were introduced to define the possible extremes of in vitro-polarized cells [43, 44].

EVs showed a major contribution to the polarization of macrophages towards TAMs, which is in agreement with previous studies showing diverse effects of EVs on macrophages [19–21]. Since many effects were induced by WT EVs derived from IMP2-expressing cells, and not by KO EVs, a central role of IMP2 in the polarization of macrophages towards an M2-like phenotype by cancer cell-derived EVs is suggested.

To further study the effect of EVs on macrophages in vivo, we used a zebrafish embryo model in which macrophages fluoresce. Zebrafish represent a favorable alternative model in biomedical research in accordance with the 3R rules. It has been reported that the plasticity and diversity of zebrafish macrophages resemble their mammalian counterparts. Therefore, it is a suitable model to study the interaction of immune cells with nanoparticles [45–47]. Our data verified the in vitro results as zebrafish macrophages, upon polarization with WT EVs, showed lower proinflammatory gene expression levels compared to KO EVs. Nevertheless, it is essential to note that studies have reported conflicting results regarding the role of IL6 in the TME. On the one hand, IL6 may sustain a pro-tumor milieu by supporting angiogenesis and tumor evasion of immune surveillance. On the other hand, IL6 has been described as opposing tumor growth by mobilizing anti-tumor T cell responses [48, 49]. In the current study, we investigated changes in IL6 expression in two different species: primary human macrophages and zebrafish embryos. Given that the biodistribution of EVs and the extent of EV uptake by cells are likely to vary between different species, it is not surprising that we observed differences in the gene expression levels.

The effects on HMDMs that we observed in this study may partly be mediated directly by vesicular IMP2, which was only detected in WT EVs. Interestingly, M2 macrophages have been shown previously to exhibit elevated IMP2 levels, suggesting that IMP2 may influence the macrophage phenotype [50]. However, our study showed that IMP2 also influences the miRNA and protein cargo of EVs. Lately, it has been shown that IMP1, another IMP family member, promoted melanoma and neuroblastoma metastases by altering the composition of EVs secreted by cancer cells [51, 52]. Since EVs facilitate much of their action *via* the delivery of miRNAs to their target cells [53–59] and IMP2 has been shown to interact with miRNA regulatory pathways [7], we hypothesized that an altered miRNA cargo might contribute to the different actions of WT and KO EVs on macrophages. To test this hypothesis, we performed miRNA sequencing with EVs and cells. Among the differentially expressed miRNAs in EVs, three miRNAs (miR-181a-5p, miR-181a-3p, and miR-452-5p) were highly abundant in WT EVs and parental cells. These miRNAs have been implicated in the development of metabolic diseases, cancer, and macrophage polarization [38, 39, 60–65]. Indrieri and co-workers demonstrated the protective effects of miR-181a/b downregulation in mitochondrial diseases [62], and miR-181a has been described as a regulator of inflammatory responses in monocytes and macrophages [60]. Very recently, others have also shown an EV-facilitated transfer of miR-181a-5p into macrophages that induces an M2-like phenotype [66]. In another study, miR-181a-5p was identified as a negative regulator in high mobility group box-1 protein-induced immune responses by targeting *TNF* mRNA [67]. miR-452-5p was identified as a regulator of pancreatic endocrine dysfunction [61]. In addition, miR-452-5p in exosomes secreted from HCC cells induced an M2 polarization, thus stimulating HCC progression by targeting tissue inhibitor of metalloprotease 3 (TIMP3) [39]. The same miRNA was upregulated in CRC and promoted the progression of CRC by activating the miR-452-PKN2/DUSP6 pathway [38]. Besides miRNAs, other EV-associated cargoes could direct macrophage polarization. Our study suggests that IMP2 influences, amongst other things, the abundance of proteins involved in metabolic processes in EVs. Further studies are required to determine whether, which, and how specific vesicular proteins may affect the macrophage phenotype and function.

Several routes of EV uptake have been proposed in macrophages as outlined in [36], and there seems to be limited agreement on the most important mechanism of EV uptake. A population of EVs can simultaneously engage multiple entry pathways into a cell, with the primary route mainly depending on the cell type [36]. In this study, we found phagocytosis and macropinocytosis to be the major routes of EV uptake. We demonstrated that lower levels of miR-181a-5p correlate with alterations in gene expression and macrophage polarization supporting multiple findings by others reporting miR-181a as important immunosuppressive and M2-inducing regulator in macrophages (e.g., [60, 66]).

We observed that the boiling process interfered with the miRNA stability and/or delivery into the cells. Although it has been claimed by Chen and co-workers that miRNAs survive the boiling process [68], others have shown miRNA degradation under various conditions such as high temperature, freeze–thaw, and humidity [69–73]. The boiling process also denatures proteins that are, on the one hand, necessary for the uptake of EVs and, on the other hand, are probably partly responsible for the effects of EVs.

We investigated the role of miR-181a-5p, which showed the highest abundance in WT EVs, on macrophage polarization. We confirmed that *DUSP6* is a direct target of miR-181a-5p, and its mRNA abundance is negatively regulated by the miRNA. miR-181a has been described to control T cell receptor activation through several phosphatases, in particular DUSP6 [74, 75]. Moreover, the importance of DUSP6 to antagonize the tumor-supporting action of ERK1/2 in colorectal cancer has been reported [37, 38].

The regulatory role of phosphatase DUSP6 in T cell metabolism towards glycolysis has been described [42]. Here we observed higher miR-181a-5p and lower *DUSP6* levels in the polarized macrophages with WT EVs, which might contribute to an EV-mediated shift towards oxidative phosphorylation (OXPHOS). However, the differences in protein cargo may also affect macrophage metabolism upon EV treatment. Further studies are needed to gather respective experimental evidence. Besides the role of DUSP6 in metabolism, changes in the phenotype of macrophages could also explain the alterations in the metabolic activity upon polarization with EVs. It has been shown that M1 macrophages have an enhanced glycolytic metabolism to fulfill their energy demands, while M2 macrophages mainly rely on OXPHOS [76, 77]. The lower basal OCR and higher ECAR values observed in macrophages polarized with KO EVs indicate a metabolic shift from OXPHOS to glycolysis, which is mainly observed in classically activated macrophages. Thus, the proinflammatory phenotype induced by KO EVs may directly cause metabolic effects. Alternatively, differentially abundant proteins in KO *vs*. WT EVs may also influence the metabolic shift. However, the contribution of specific proteins to the metabolic shift observed in EV-treated macrophages remains to be elucidated.

The limitation of our current study lies in the utilization of a single type of colon cancer cell line, namely HCT116 WT and KO. It is important to note that these cells closely mirror the clinical context, as recently highlighted by Kendzia and colleagues [6]. In fact, our group has made numerous attempts to generate IMP2 KO cells using diverse methods ([22] and data not shown). However, these efforts typically resulted in non-proliferating cells, indicating the importance of IMP2 for cell proliferation. Additionally, by showing a reduction in expression of miR-181a-5p in three independent IMP2 KO cell clones, the observed effects are unlikely to be a clonal artifact.

## Conclusion

Our results indicate that EVs secreted from IMP2-expressing HCT116 cells contribute to a polarization of macrophages towards a TAM-like phenotype. This polarization strongly depends on IMP2 expression, which alters the composition of vesicular miRNAs and proteins. The IMP2-dependent changes in EV composition affect tumor-suppressive signaling pathways and, most likely, contribute to an altered macrophage metabolism. In summary, we showed that IMP2 in cancer cells can modulate the influence of cancer cell-derived EVs on macrophages, which may contribute to tumor progression.

### Supplementary Information

Supplementary Material 1.

Supplementary Material 2.

## Data Availability

The miRNA-Seq raw and processed data were deposited in the Gene Expression Omnibus repository (GSE235115). The mass spectrometry proteomics data have been deposited to the ProteomeXchange Consortium *via* the PRIDE partner repository with the dataset identifier PXD052287.

## Abbreviations

ABCG1: ATP binding cassette transporter G1
ALIX: Programmed cell death 6-interacting protein (PDCD6IP)
BCA: Bicinchoninic acid
CD: Cluster of differentiation
cDNA: Complementary DNA
CRC: Colorectal cancer
CRISPR: Clustered regularly interspaced short palindromic repeats
DMEM: Dulbecco’s Modified Eagle’s Medium
dpf: Days post fertilization
dpi: Days post injection
DUSP6: Dual specificity phosphatase 6
ECAR: Extracellular acidification rate
eef1a: Elongation factor 1 alpha
eGFP: Enhanced green fluorescent protein
EIPA: 5-(N-Ethyl-N-isopropyl)-Amiloride
ELISA: Enzyme‑linked immunosorbent assay
EVs: Extracellular vesicles
FACS: Fluorescence-activated cell sorting
FCCP: Carbonyl cyanide-p-trifluoromethoxy-phenylhydrazone
FSC: Forward scatter
HCC: Hepatocellular carcinoma
HIF1A: Hypoxia-inducible Factor 1 alpha
HLA: Human leukocyte antigen complex
HMDMs: Human monocyte-derived macrophages
hpf: Hours post fertilization
hpi: Hours post-injection
HSP70: Heat shock protein 70
IGF2BP2/IMP2: Insulin like growth factor 2 mRNA binding protein 2
IL6: Interleukin 6
IL1B: Interleukin 1 beta
KEGG: Kyoto Encyclopedia of Genes and Genomes
KO: Knockout
MAPK: Mitogen-activated protein kinase
M-CSF: Macrophage colony-stimulating factor
MFI: Median fluorescence intensity
miRNA: MicroRNA
MISEV: Minimal information for studies of extracellular vesicles
MMP9: Matrix metalloproteinase 9
mpeg: Macrophage‐expressed gene
mPES: Modified polyethersulfone
MTT: 3-(4,5-Dimethylthiazol-2-yl)-2,5-diphenyltetrazolium bromide
NTA: Nanoparticle tracking analysis
NTC: Non-target control
OCR: Oxygen consumption rate
OXPHOS: Oxidative phosphorylation
PBMC: Peripheral blood mononuclear cells
PCA: Principal component analysis
PI: Propidium iodide
PTU: Propylthiouracil
qPCR: Real-time quantitative polymerase chain reaction
RPMI: Roswell Park Memorial Institute
SD: Standard deviation
SFM: Serum-free medium
SSC: Side scatter
TAMs: Tumor-associated macrophages
TCM: Tumor cell conditioned medium
TEM: Transmission electron microscopy
TFF: Tangential Flow Filtration
TME: Tumor microenvironment
TNF: Tumor necrosis factor
TSG: Tumor susceptibility gene
UC: Ultracentrifugation
VISTA: V domain-containing Ig suppressor of T-cell activation
VSIR: V-set immunoregulatory receptor
WB: Western blot
WT: Wildtype
