# Supplementary material for: The RNA binding protein IGF2BP2/IMP2 alters the cargo of cancer cell-derived extracellular vesicles supporting tumor-associated macrophages
Source: Cell Commun Signal. 2024 Jun 27;22:344. doi: 10.1186/s12964-024-01701-y (PMC11212187; doi:10.1186/s12964-024-01701-y)
Supplement: Supplementary file 1 — Supplementary Material 1. [file 12964_2024_1701_MOESM1_ESM.docx]

**Supplementary information**

**
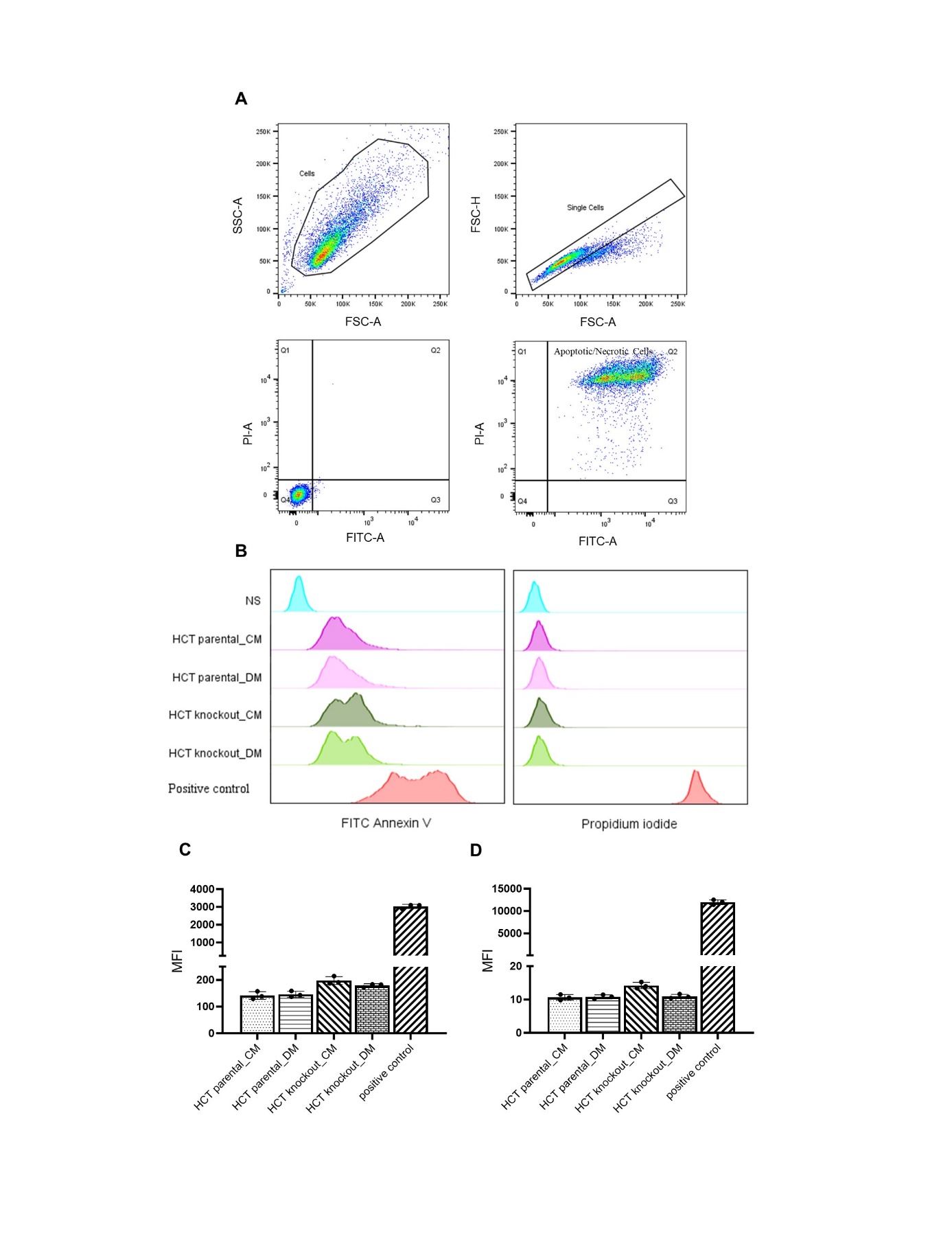
**

**Fig. S1** HCT116 cells are neither apoptotic nor necrotic in EV-depleted media. Cells were cultured in complete or EV-depleted media for 48 h and then the viability was measured with Annexin V/PI staining using flow cytometry. **A** Gating strategy was applied for the analysis. **B** Representative histograms. **C** Quantification of apoptotic and **D** necrotic cells. NS: non-stained; CM: complete medium; DM: depleted medium; MFI: median fluorescence intensity. Negative control: non-stained HCT cells; Positive control: HCT cells heated at 60 °C for 20 min. Data are shown as mean ± SD (n=3, triplicates).

**
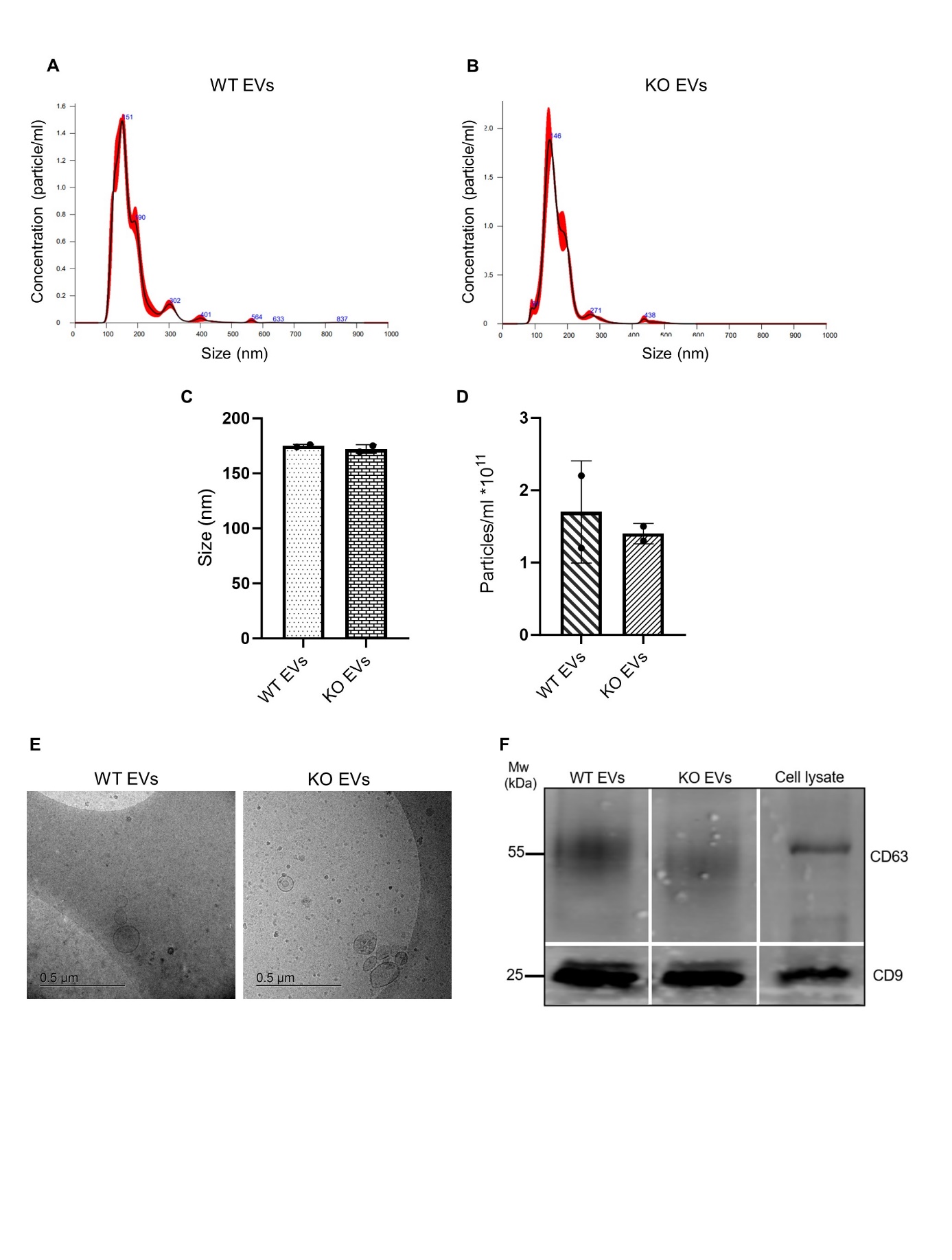
**

**Fig. S2** Characterization of HCT116 parental and knockout cell-derived EVs isolated *via* the UC method. **A, B** Representative NTA size distribution profiles of isolated EVs. **C, D** NanoSight quantification of EV preparations’ size (C) and concentration (D) are shown as mean ± SD, n=2 individual EV isolations, triplicates. **E** Cryo-TEM images of EVs, scale bar: 500 nm. **F** The expression of EV markers (CD63, CD9) was determined by Western blot analysis. Lanes from left to right; WT EVs, KO EVs, and HCT116 cell lysate.

**
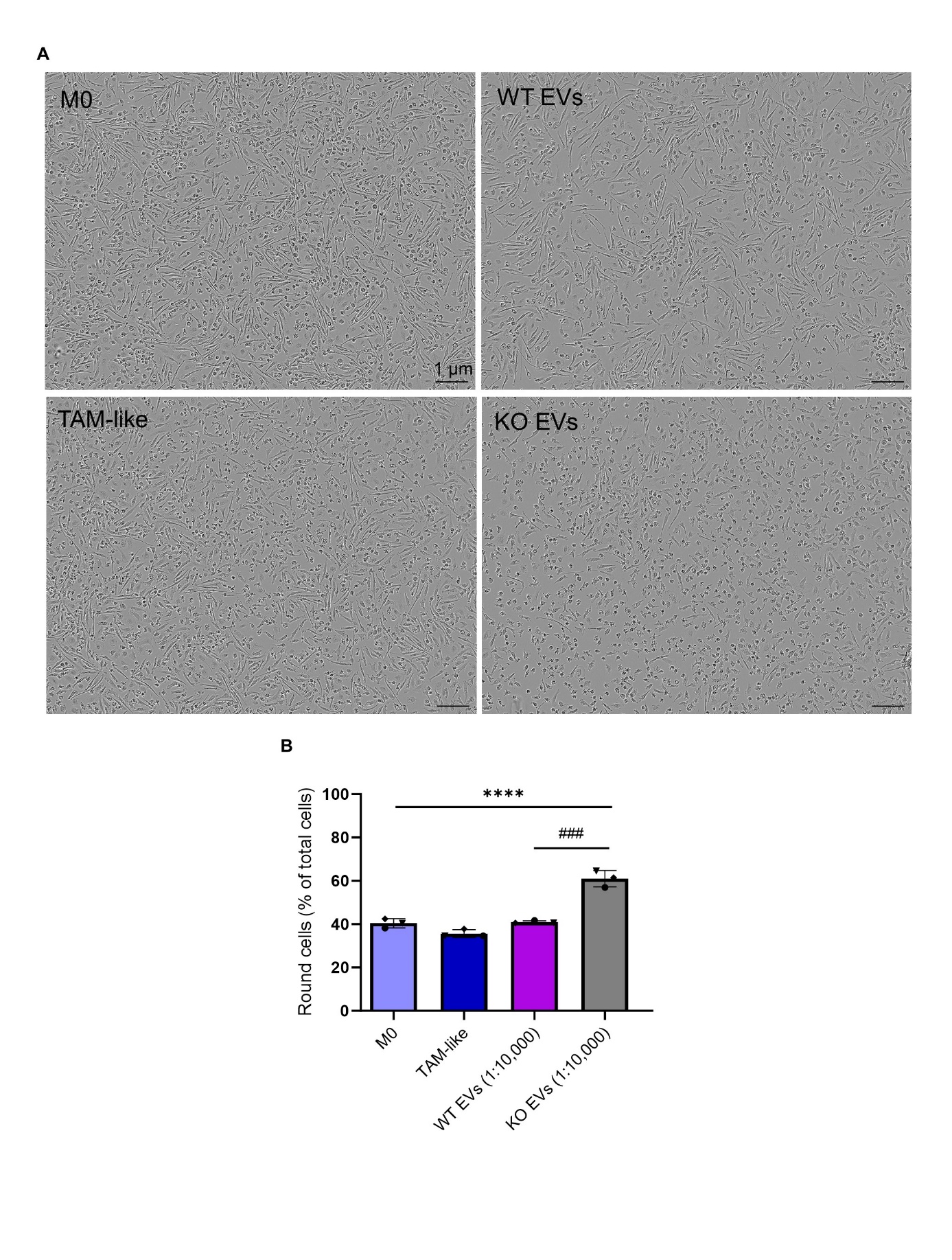
**

**Fig. S3** Macrophages polarized with KO EVs show a more pronounced M1-like phenotype. Cells were grouped based on their eccentricity in a round or elongated phenotype by the Incucyte cell-by-cell analysis software module. **A** Representative images of macrophages, either non-polarized or polarized for 24 h with TCM (TAM-like) or EVs that were isolated with the UC method, are shown. **B** Percentage of macrophages with round morphology (n=3 individual donors, triplicates). Statistical analysis was performed using one-way ANOVA followed by Bonferroni’s post-hoc test. Data are shown as mean ± SD, and p<0.05 is considered significant. * indicates a significant difference between macrophage treatments and M0. # shows a significant difference between WT and KO EVs.

**
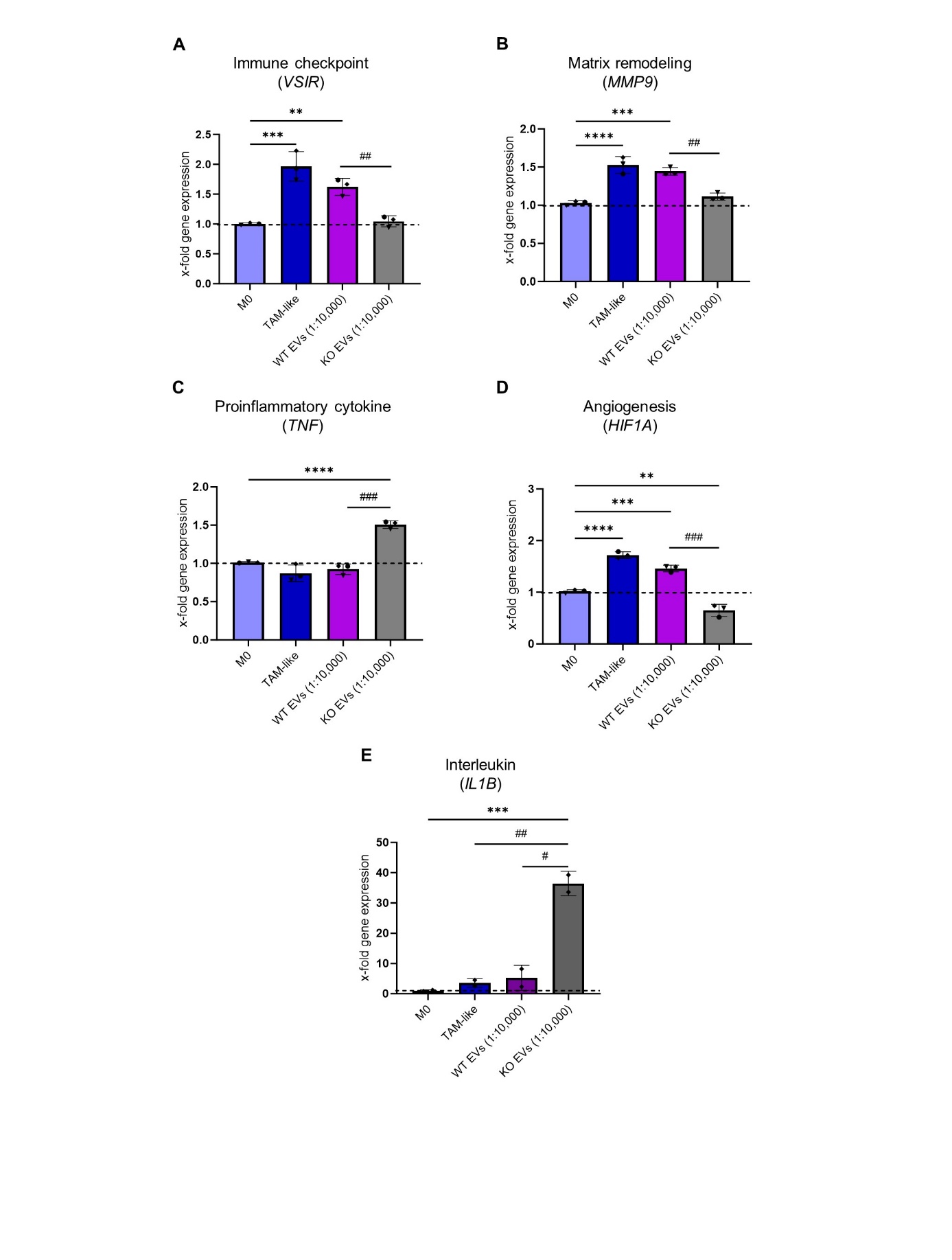
**

**Fig. S4** WT EVs polarize macrophages towards a TAM-like phenotype. Changes in gene expression in the non-polarized M0 and polarized macrophages were assessed by qPCR. **A-D** Primary macrophages were polarized with EVs isolated with the UC method at a ratio of 1:10,000 (cell:EV), and TCM (TAM-like) for 24 h and the expression of *VSIR*, *MMP9*, *TNF*, and *HIF1A* was quantified (n=3 individual donors, triplicates). **E** Primary macrophages were polarized with EVs isolated with the TFF method at a ratio of 1:10,000 (cell:EV), and TCM (TAM-like) for 24 h and the expression of *IL1B* was quantified (n=2 individual donors, duplicates). Statistical analysis was performed using one-way ANOVA followed by Bonferroni’s post-hoc test. Data are shown as mean ± SD, and p<0.05 is considered significant. * indicates a significant difference between treatments and M0. # shows a significant difference between WT and KO EVs.

**
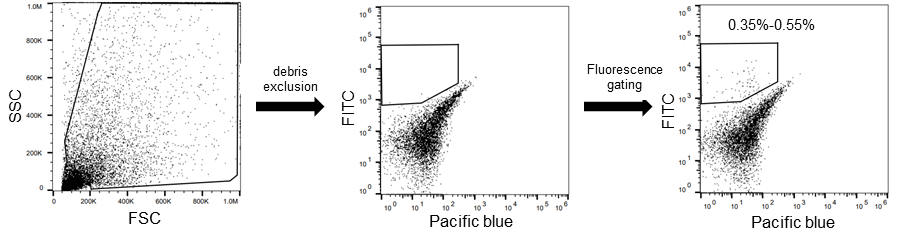
**

**Fig. S5** Gating strategy for sorting zebrafish macrophages. eGFP^+^ cells were sorted by FACS analysis. The FITC channel was used for positive macrophages and the Pacific blue channel was used for non-fluorescent cells.

**
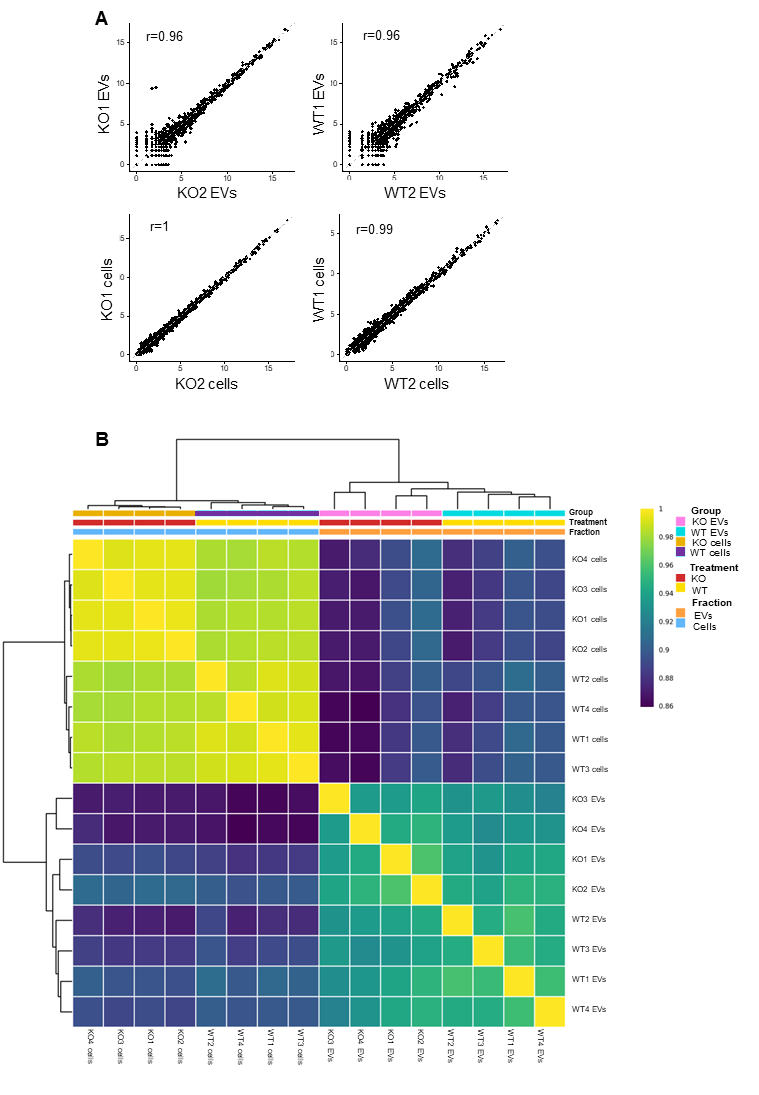
**

**Fig. S6** Overview of miRNA correlation between EVs and cells. **A** Representative scatter plots showing the correlation of miRNA profiles between the replicates of EVs and cells. **B** A high correlation was observed among the replicates of each group. The largest separation was observed between EVs and cells and then between conditions inside each fraction. In the heatmap, the higher the number, the better the correlation is between the replicates.

**
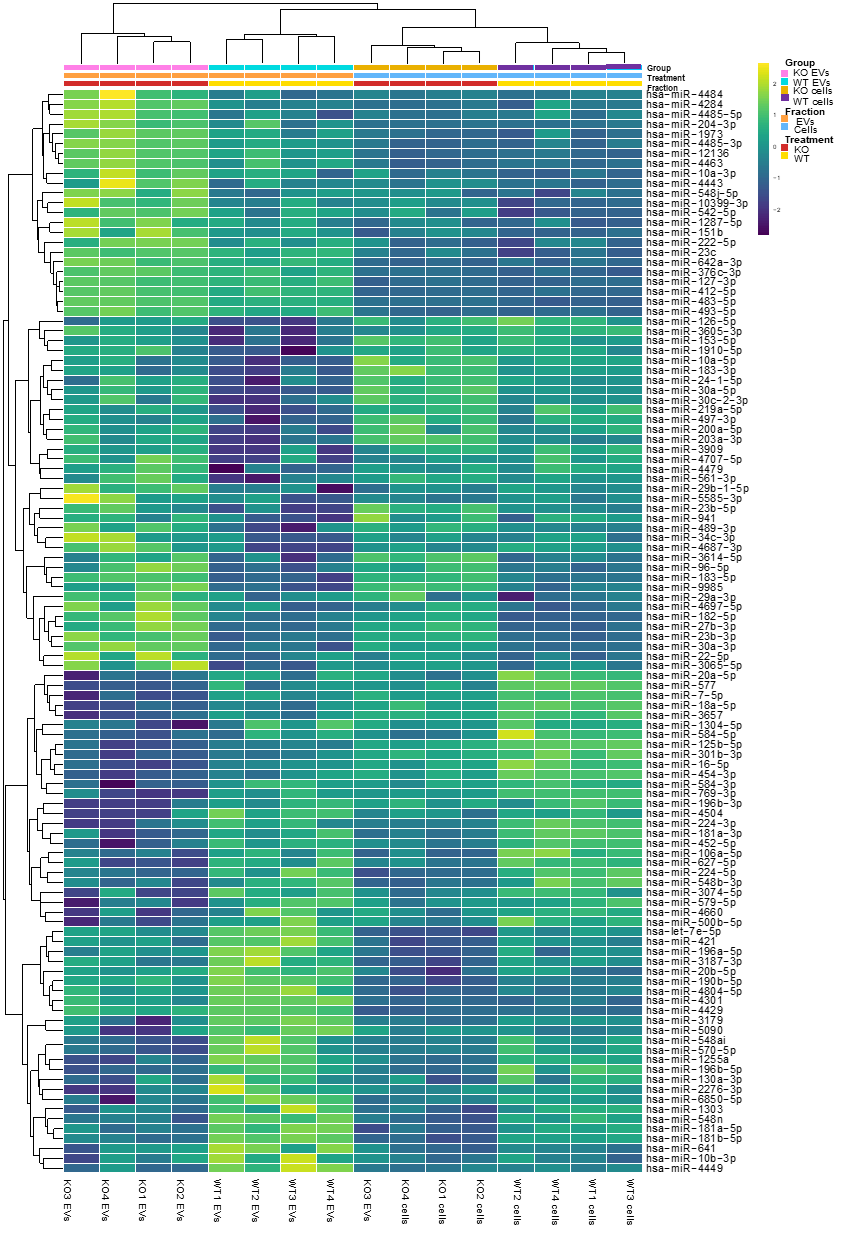
**

**Fig. S7** Differentially expressed miRNAs in EVs and cells. The heatmap represents the hierarchical clustering of miRNAs that are expressed in EVs and cells. The positive values indicate higher expression in WT EVs and parental cells, the negative values indicate higher expression in KO EVs and knockout cells, and 0 shows no difference in the expression of miRNAs.

**Table S1** List of miRNAs highly expressed in WT EVs

| miRNA | log_2_FC | p-value | RPM |
| --- | --- | --- | --- |
| hsa-let-7e-5p | -0.860948671 | 0.001448525 | 7917.465833 |
| hsa-miR-106a-5p | -0.342859774 | 0.019027511 | 76.40658266 |
| hsa-miR-10b-3p | -1.016671527 | 0.047746392 | 7.634206481 |
| hsa-miR-1255a | -1.438561727 | 0.001352519 | 32.47678912 |
| hsa-miR-125b-5p | -0.656748809 | 0.041183591 | 158.4150276 |
| hsa-miR-1303 | -1.30941089 | 0.015958635 | 64.67150461 |
| hsa-miR-1304-5p | -1.307232393 | 0.035251802 | 5.185779599 |
| hsa-miR-130a-3p | -0.255199128 | 0.044160724 | 3100.906581 |
| hsa-miR-16-5p | -0.520023234 | 0.00493702 | 9235.804711 |
| hsa-miR-181a-3p | -1.552598681 | 0.031084794 | 45.13661784 |
| hsa-miR-181a-5p | -1.450467451 | 0.000937087 | 7330.932616 |
| hsa-miR-181b-5p | -1.722331408 | 0.000141694 | 3712.767965 |
| hsa-miR-18a-5p | -0.565552122 | 0.01884288 | 3401.582715 |
| hsa-miR-190b-5p | -0.956631431 | 0.006423175 | 29.47122698 |
| hsa-miR-196a-5p | -0.919004678 | 0.018932774 | 2998.85658 |
| hsa-miR-196b-3p | -1.632595432 | 0.003562058 | 0.854509005 |
| hsa-miR-196b-5p | -1.418305565 | 0.000197036 | 3007.363102 |
| hsa-miR-20a-5p | -0.615159171 | 0.039927068 | 9962.193591 |
| hsa-miR-20b-5p | -0.369049225 | 0.010598405 | 34.86036104 |
| hsa-miR-224-3p | -1.567951748 | 0.026086573 | 2.106799454 |
| hsa-miR-224-5p | -1.639686571 | 0.009730656 | 83.22181464 |
| hsa-miR-2276-3p | -1.423200797 | 0.018420416 | 1.921072296 |
| hsa-miR-301b-3p | -0.46647502 | 0.024061759 | 352.4773374 |
| hsa-miR-3074-5p | -2.188551511 | 0.031423996 | 3.180753943 |
| hsa-miR-3179 | -1.957528901 | 0.032597931 | 11.11326346 |
| hsa-miR-3187-3p | -1.171981865 | 0.032544434 | 121.988678 |
| hsa-miR-3657 | -0.832312207 | 0.017112619 | 20.09052772 |
| hsa-miR-421 | -0.6653334 | 0.007253948 | 114.8668916 |
| hsa-miR-4301 | -1.866555971 | 0.016179462 | 14.89653116 |
| hsa-miR-4429 | -1.071314149 | 0.017863843 | 44.28372477 |
| hsa-miR-4449 | -3.263699767 | 0.001874248 | 3.191716421 |
| hsa-miR-4504 | -2.152437187 | 0.01938515 | 2.492911846 |
| hsa-miR-452-5p | -2.843304656 | 0.022068558 | 12.43030655 |
| hsa-miR-454-3p | -0.547284816 | 0.018653407 | 464.6002487 |
| hsa-miR-4660 | -2.025776647 | 0.045722306 | 4.060176169 |
| hsa-miR-4804-5p | -1.075644945 | 0.037332694 | 46.28036241 |
| hsa-miR-500b-5p | -0.726519505 | 0.011086334 | 75.37967717 |
| hsa-miR-5090 | -1.879208133 | 0.043869457 | 2.799162451 |
| hsa-miR-548ai | -1.019821758 | 0.008229672 | 10.22122181 |
| hsa-miR-548b-3p | -1.387116039 | 0.028443866 | 4.654741274 |
| hsa-miR-548n | -1.96964436 | 0.000697026 | 16.76752017 |
| hsa-miR-570-5p | -1.019821758 | 0.008229672 | 10.22122181 |
| hsa-miR-577 | -0.827697021 | 0.039151261 | 23.71664298 |
| hsa-miR-584-3p | -1.780613346 | 0.013278757 | 13.20766521 |
| hsa-miR-584-5p | -0.441583346 | 0.049978898 | 146.8043571 |
| hsa-miR-627-5p | -0.726674289 | 0.021218804 | 199.6330983 |
| hsa-miR-641 | -0.751726692 | 0.026375093 | 374.8666363 |
| hsa-miR-6850-5p | -3.033743012 | 0.016871208 | 14.82059712 |
| hsa-miR-7-5p | -1.41104443 | 0.005754461 | 15777.52243 |
| hsa-miR-769-3p | -0.868304333 | 0.020249201 | 119.9062875 |

**
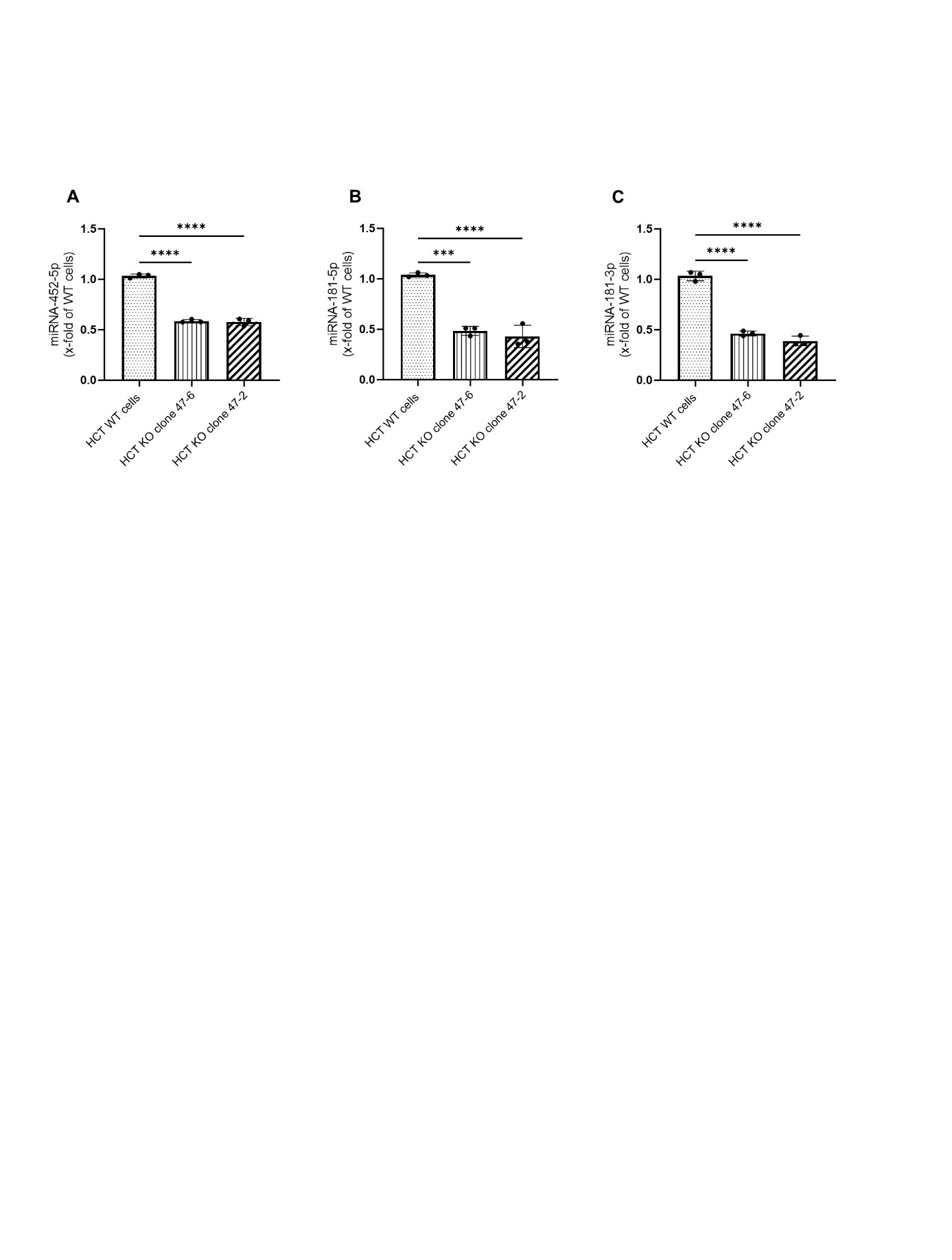
**

**Fig. S8** KO clones show similar miRNA content. Differentially expressed miRNAs (miR-452-5p, miR-181a-5p, and miR-181a-3p) were assessed in HCT116 KO clones (KO-47-2 and 47-6) by qPCR using miRCURY LNA SYBR Green PCR kit. The content of miRNAs was normalized to U6 and is shown as a fold change relative to WT cells. Statistical analysis was performed using one-way ANOVA followed by Bonferroni’s post-hoc test. Data are presented as mean ± SD, n=3, triplicates. * indicates a significant difference between treatments and M0.


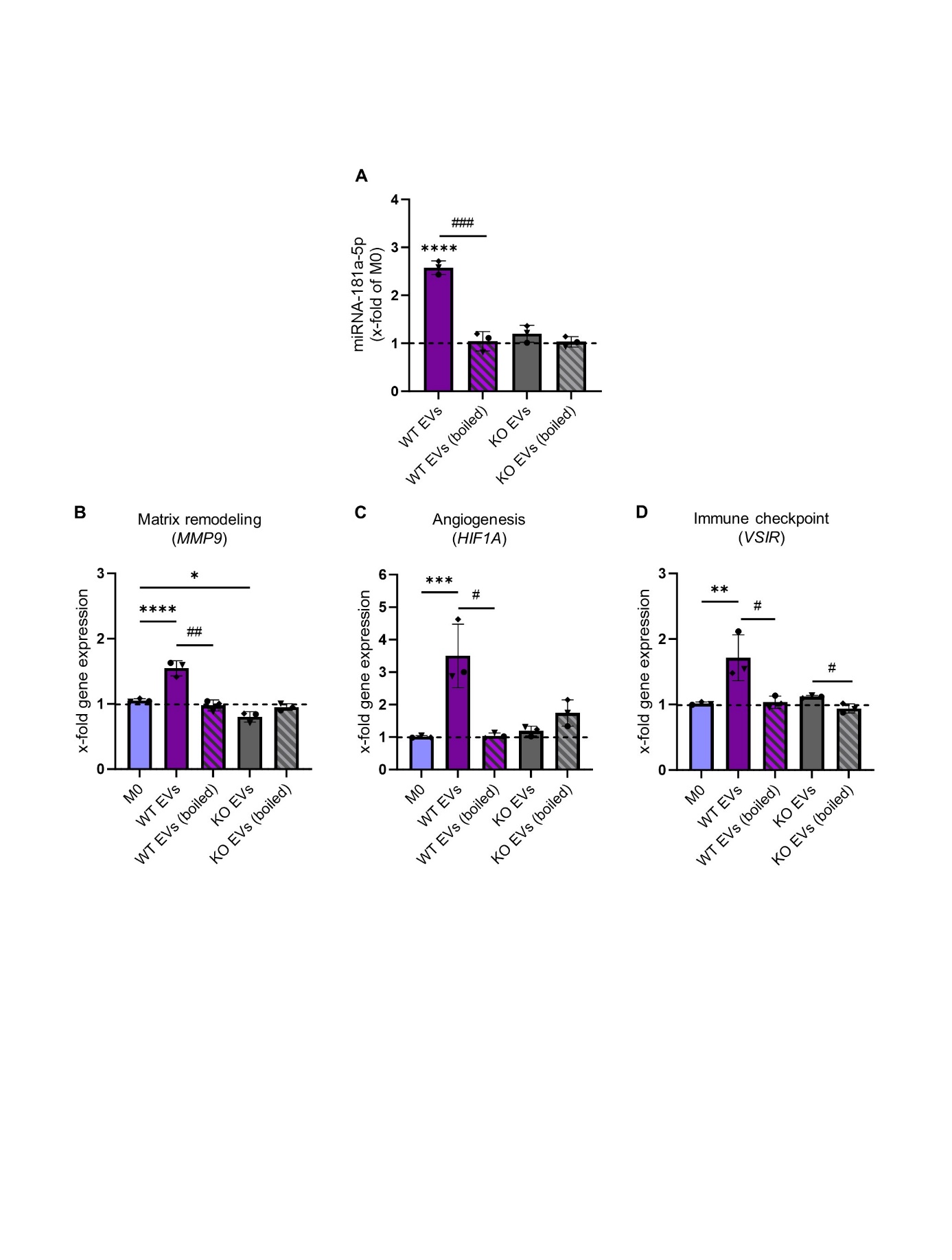


**Fig. S9** Boiled EVs fail to deliver miRNA into macrophages. **A** The amount of miR-181a-5p was quantified by qPCR in cells that were treated with boiled and non-boiled EVs (1:20,000 cell:EV ratio) for 24 h (n=3 individual donors, triplicate). **B-D** Changes in the expression of *MMP9*, *HIF1A*, and *VSIR* were assessed by qPCR. Statistical analysis was performed using one-way ANOVA followed by Bonferroni’s post-hoc test. Data are shown as mean ± SD, and p<0.05 is considered significant. * indicates a significant difference between treatments and M0. # shows a significant difference between boiled and non-boiled EVs.


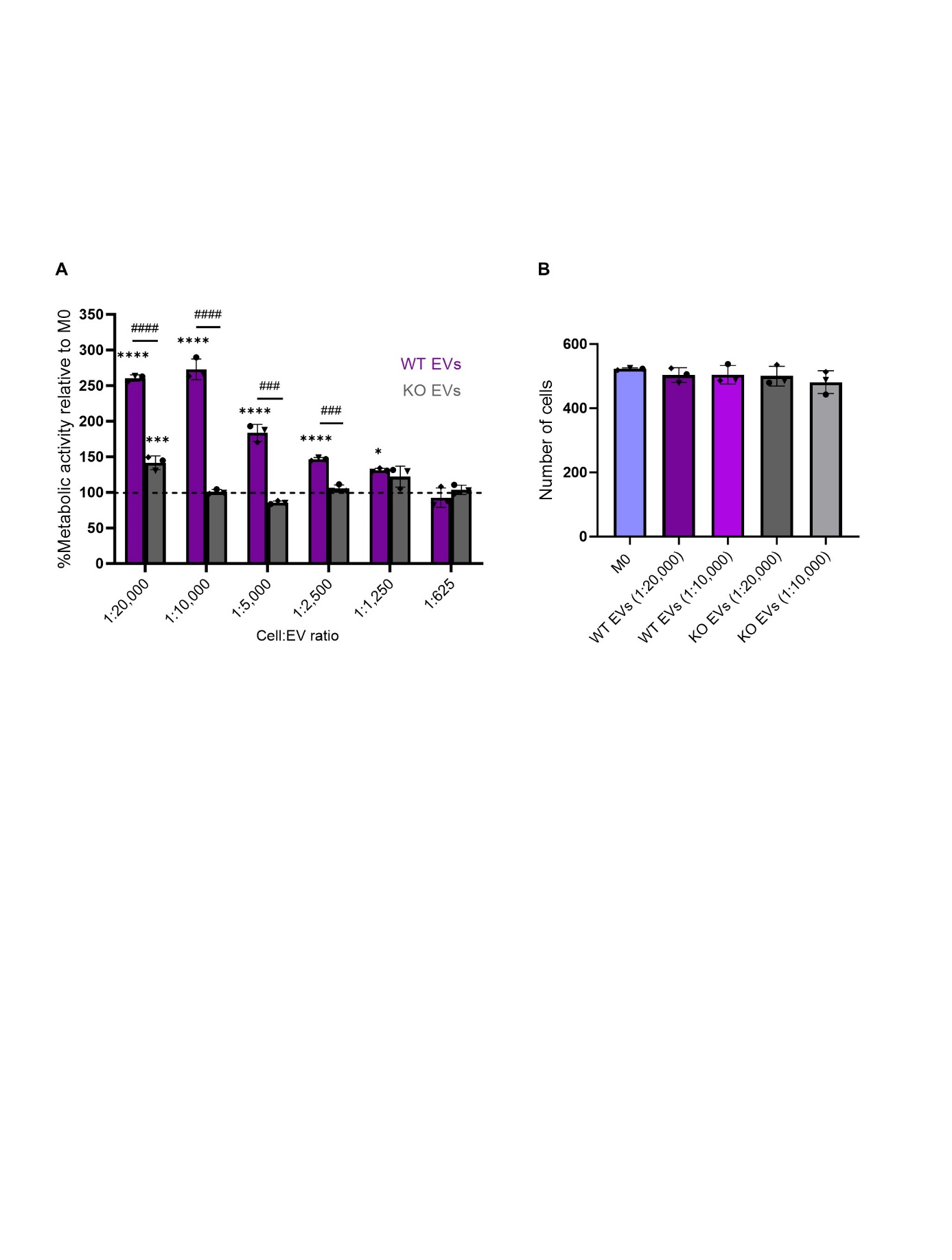


**Fig. S10** EVs affect macrophage metabolism. **A** *In vitro* metabolic activity of primary macrophages was measured in an MTT assay after 24 h polarization with EVs isolated with the UC method (n=3 individual donors, triplicates). **B** Number of cells does not change after polarization with EVs. The number of macrophages was counted with the Incucyte cell-by-cell analysis software module. Statistical analysis was performed using one-way ANOVA followed by Bonferroni’s post-hoc test. Data are shown as mean ± SD, and p<0.05 is considered significant. * indicates a significant difference between treatments and M0. # shows a significant difference between WT and KO EVs.
